# Forces in mechanically interlocked materials

**DOI:** 10.1039/d6cs00328a

**Published:** 2026-07-21

**Authors:** Narangerel Ganbaatar, Xun Li, James Ormson, Valentin Foidart, Guillaume De Bo, Charles-André Fustin, Anne-Sophie Duwez

**Affiliations:** a Department of Chemistry, UR MolSys, University of Liège 4000 Liège Belgium asduwez@uliege.be; b Department of Chemistry, University of Manchester Oxford Road Manchester M13 9PL UK guillaume.debo@manchester.ac.uk; c Institute of Condensed Matter and Nanosciences (IMCN), BSMA, Université catholique de Louvain 1348 Louvain-la-Neuve Belgium charles-andre.fustin@uclouvain.be

## Abstract

Forces are at the heart of almost every major biological process. The widespread use of molecular machines in nature has inspired scientists to synthesize mechanically interlocked molecules (MIMs) in which controlled, relatively large amplitude motion of one component relative to another can potentially result in net directional forces. This review examines force-related processes in MIMs, namely, catenanes, rotaxanes and knots, and in the polymers that include them. We first discuss the single-molecule force spectroscopy studies, performed on well-defined mechanically linked systems, one molecule at a time, and show how these experiments have provided unprecedented insights into their operation, dynamics, and comprehensive understanding of their performance metrics. Then the use and effects of these mechanical links on mechanically activated polymers are addressed. We examine the unique mechanochemical reactivity of MIMs and how it is exploited to create force-responsive molecular devices and materials or elicit new mechanochemical reactions in response to external force. Finally, we focus on the use of mechanical links as crosslinks, giving rise to the so-called slide-ring materials. The review highlights the unprecedented mechanochemical properties that are emerging from the integration of MIMs into polymers and the quick pace of progress in the field that should give rise to more advanced systems.

## Abstract 2

This review highlights force-driven processes in mechanically interlocked molecules and polymers, with a focus on insights from single-molecule studies, mechanochemistry, force-responsive materials, and slide-ring systems.

## Graphical abstract

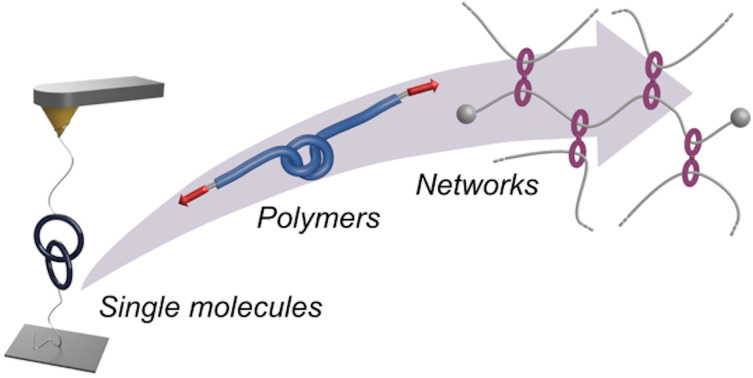

## 1. Introduction

Forces and mechanical stress are omnipresent in biology. Cells, for example, are a very turbulent environment, subject to numerous mechanical deformations and shear forces. Mechanical activation of chemical bonds is commonly observed in nature. Many physiological processes rely on strained molecular structures, such as enzymatic activity, the functioning of molecular motors, cell division, and muscle contraction.^1^ Forces are also widely present in everyday materials, like polymer cloths and textiles. However, in biomedicine or materials science applications, the bonds are still chosen on the basis of their thermodynamic binding constant, not for their mechanical stability, although it is now well accepted that the thermodynamic stability may be very different from the mechanical stability. Bonds considered to be very weak can turn out to be mechanically robust, and in contrast, strong bonds can turn out to be fragile when subjected to a mechanical load.^2,3^

**Fig. 1:**
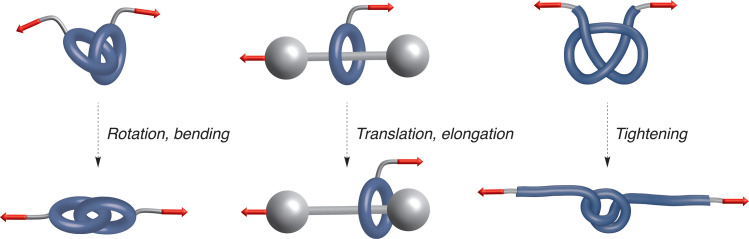
Schematic representation of the main force-induced submolecular motions in a catenane, a rotaxane, and a knot.

Mechanically interlocked molecules (MIMs) are molecules in which multiple components are held together by mechanical bonds, *i.e.*, the components are entangled in a way that prevents their separation without breaking a covalent bond. Unlike molecules connected by standard chemical bonds, MIMs rely on particular topologies for their connection.^4^ As an external mechanical force is applied, they respond by various movements (rotation, translation, bending, elongation, *etc.*) before the rupture of covalent bonds occurs at high force (Fig. 1). These responses give rise to singular behaviours, opening the way to materials with unique properties.

In 1940, Kauzmann and Eyring^5^ proposed that mechanical forces within polymer networks could cause covalent bond scission, ultimately leading to the fracture and failure of polymer materials. The force driving chain fracture was identified as a key factor in determining material fracture toughness, as highlighted by Lake and Thomas.^6^ More recently, studies on how mechanical forces couple with bond scission have revealed new material responses, including self-strengthening and damage sensing. However, the mechanochemical role of topology—such as knots and mechanically interlocked architectures like rotaxanes (a dumbbell-shaped axle which is threaded through a macrocycle) and catenanes (two or more interlocked macrocycles)—remains less well understood. As the use of mechanically linked polymers expands, understanding these interactions is becoming increasingly important. Moreover, mechanically interlocked motifs such as catenanes may also serve as useful models for probing the behaviour of strong entanglements, which are otherwise challenging to study directly.

Here, we review force-related processes in MIMs, namely, catenanes, rotaxanes and knots, and in the polymers that include them. The first section deals with well-defined molecules, containing only one or a limited number of mechanical links, studied at the level of individual molecules by single-molecule force spectroscopy (SMFS). In the second section, we discuss the use and effects of these mechanical links on mechanically activated polymers. The last section focuses on the use of these mechanical links as crosslinks, giving rise to the so-called slide-ring materials.

## 2. Measuring forces in mechanically interlocked molecules, one at a time

Single-molecule force spectroscopy consists in trapping and stretching an individual molecule with a device to probe molecular processes *in situ* and in real time through the application of mechanical forces. The device can be the tip of an atomic force microscope (AFM), a laser trap, or magnetic beads. Because of the low trap stiffness, the range of forces accessible with optical tweezers (OT) and magnetic tweezers is lower in comparison with that of AFM. Optical tweezers and magnetic tweezers offer better temporal and force resolution, in addition to the possibility of exerting torque, while AFM offers a better spatial resolution. During the last two decades, SMFS has demonstrated its effectiveness in providing mechanistic insights into biomolecules and in quantifying how they respond to external mechanical forces. Two main approaches are used: (i) molecular recognition in which the two partners of a bond or a molecular complex are put into contact and then pulled apart or (ii) the pulling of a single molecule in which intra-molecular processes take place.

SMFS studies have provided unprecedented insights into the structure and function of numerous biological systems, including proteins, DNA or RNA, and biomolecular machines.^7–19^ In addition to its extensive application in biological research, this technique has also been adapted to investigate intramolecular processes in synthetic macromolecules such as polymers,^20^ mechanophores,^21–25^ and mechanically interlocked systems.^26,27^ However, studies focusing on intramolecular processes and the mechanical behaviour of small molecules (1–5 nm) remain relatively scarce. Notable examples include investigations of molecular recognition pairs,^28^ helical architectures,^29^ and prototypes of artificial molecular machines.^30–36^ The limited number of such studies mainly arises from challenges associated with designing suitable molecular systems and developing experimental tools that allow effective interfacing with SMFS techniques, particularly when probing motions at the submolecular scale. Given the very small scale of the involved processes, the medium made of organic solvent and the relatively high forces involved, AFM is the most suitable technique to carry out the SMFS experiments on those systems, unless molecules are modified to be compatible with other techniques.

While SMFS provides direct access to the forces associated with molecular interactions and transformations, interpreting these forces in terms of intrinsic interaction strengths is not straightforward. In particular, the relationship between measured rupture forces and the underlying bonding motifs is influenced by a range of experimental and molecular parameters, making it essential to place the observed values within an appropriate physical framework. Historically, relatively well-defined force ranges were often associated with specific classes of molecular interactions. However, it has become increasingly clear that these ranges can overlap significantly, owing to the strong dependence of measured rupture forces on experimental and molecular factors such as solvent, loading rate, pulling geometry, as well as linker nature, architecture and length. Importantly, even for a given type of interaction, the measured rupture force can vary by up to an order of magnitude depending on these parameters. In addition, interactions that are thermodynamically strong may exhibit comparatively low mechanical stability, and conversely, thermodynamically weaker interactions can sustain relatively high forces. This apparent discrepancy arises primarily from the anisotropic nature of force: the rupture force depends critically on the direction along which the interaction is loaded, making the pulling geometry a key determinant of mechanical response. The magnitude of forces required to disrupt molecular interactions therefore depends strongly on these parameters, and the values reported should be considered as typical orders of magnitude rather than absolute constants. Nevertheless, an approximate hierarchy of interaction strengths can still be identified. Within this context, the forces associated with different bonding motifs span a wide range, reflecting the diversity of interactions encountered in supramolecular and covalent systems. Weak noncovalent interactions such as van der Waals and π–π stacking interactions typically rupture at forces on the order of a few tens of piconewtons (∼10–50 pN), depending on geometry and loading conditions. Hydrogen bonds generally exhibit higher mechanical stability, with rupture forces typically ranging from ∼20 to 100 pN for individual interactions, although cooperative networks can sustain significantly larger forces. Metal–ligand coordination bonds cover a broader range, with rupture forces commonly between ∼30 and 300 pN depending on the metal center, coordination environment, and pulling geometry. Covalent bonds are substantially stronger, typically requiring forces in the range of ∼1–3 nN to induce rupture at standard SMFS loading rates, although this value can vary depending on the bond type, linker used to connect the bond, and loading direction. Dynamic covalent bonds, which combine covalent character with reversibility, occupy an intermediate regime: while their rupture forces can approach those of conventional covalent bonds under certain conditions, they can rupture at significantly lower forces. It is also important to note that, in SMFS experiments, access to truly isolated interactions remains rare. Instead, the measured response most often reflects the rupture of a molecular motif in which several interactions contribute collectively, resulting in an effective force that integrates these multiple contributions.

### 2.1. Forces in pseudorotaxanes and rotaxanes

#### 2.1.1 Dethreading in poly-pseudorotaxanes

Rotaxanes and pseudorotaxanes share a common structural motif in which a macrocycle is threaded onto a molecular axle. In pseudorotaxanes, however, the axle lacks bulky end groups that would act as stoppers. As a result, the macrocycle can pass over the ends of the axle and dissociate by dethreading. The first force spectroscopy investigations concerned the ability of a bulky group to stop a macrocycle from dethreading. Various experimental^37–44^ and computational^37,39,45^ studies had focused on this possibility.

**Fig. 2:**
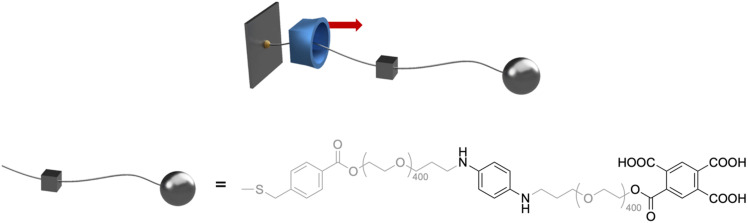
Schematic representation of the AFM force spectroscopy experiment used to probe the deslipping of α-cyclodextrin (in blue) over a tricarboxylic acid benzoyl stopper along a PEG chain. The red arrow denotes the direction of the applied pulling force.

Cyclodextrins are known to form polyrotaxanes through the threading of macrocycles onto poly(ethylene glycol) (PEG) chains.^46^ The sliding and dethreading behaviour of an α-cyclodextrin (αCD) on a PEG thread was investigated using AFM-based single-molecule force spectroscopy (Fig. 2).^47^ The force–extension curves recorded at loading rates on the order of 10 nN s^−1^—typical value for such experiments—displayed a rupture peak at a relatively low force of approximately 110 pN. For each rotaxane examined, the rupture length corresponded to the combined length of the PEG track and the tether connecting the cyclodextrin to the AFM tip. Polyrotaxanes with similar structures but varying thread lengths exhibited consistent trends. Control experiments further demonstrated that direct detachment of the polymer from the tip occurs at significantly higher forces. These observations indicate that rupture most likely occurs when the cyclodextrin slips over the bulky tricarboxylic acid benzoyl stopper, leading to dethreading of the macrocycle.

A few years later, a cyclodextrin-based pseudorotaxane was employed to quantify the strength of a calcium-mediated egg-box junction, corresponding to cross-links formed between oligoguluronic acid sequences.^48^ In these experiments, an AFM tip was used to capture an α-cyclodextrin, which was then pulled to unzip the egg-box structure. This approach enabled the determination of the force required for the cyclodextrin to slide along a single oligoguluronic acid strand. Sliding over an individual guluronic acid unit required a force of approximately 50 pN, whereas unzipping the junction required forces of about 60, 110, and 140 pN for oligoguluronic acid chains containing 2, 4, and 8 Ca^2+^ ions, respectively. Interestingly, the strength of the first cross-link depended on the number of subsequent cross-links. Analysis of the force–extension curves further indicated that the cyclodextrin did not disrupt the complexes sequentially; instead, clusters of approximately four Ca^2+^ ions were broken simultaneously. The sliding and deslipping behaviour of α-cyclodextrins was also exploited to locate specific functional groups along a polymer chain, an approach termed “sliding contact force spectroscopy”.^49^

#### 2.1.2. Estimation of the energy that could be produced by a rotaxane

**Fig. 3:**
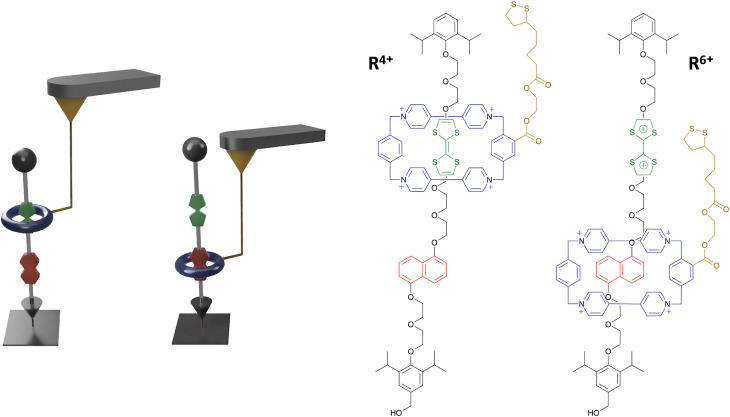
Principle of the AFM force spectroscopy experiment (left) and chemical structure of bistable [2]rotaxane R^4+^ (right). The system consists of an electron-deficient CBPQT^4+^ ring (in blue) mechanically interlocked with a molecular axle bearing two electron-rich recognition units, TTF (in green) and DNP (in red). Bulky 2,6-diisopropylphenyl ether stopper groups located at both ends of the axle prevent dethreading of the ring. The stopper adjacent to the DNP site is functionalized with a hydroxymethyl group, enabling subsequent grafting onto silicon substrates. The CBPQT^4+^ ring is equipped with a very short tether terminated by a thioctic acid ester, which allows attachment to a gold-coated AFM tip. In the reduced state, the stronger affinity of CBPQT^4+^ for TTF relative to DNP localizes the ring preferentially on the TTF station. Upon chemical oxidation of TTF to TTF^2+^, pronounced electrostatic repulsion develops between the ring and the oxidized unit, triggering shuttling of the CBPQT^4+^ ring toward the DNP site in oxidized [2]rotaxane R^6+^.

The first attempt to measure forces in rotaxanes was made by Stoddart, Houk, Ho, and co-workers.^50^ The system investigated consists of a redox-responsive, bistable [2]rotaxane (denoted R^4+^ in Fig. 3), in which an electron-deficient cyclobis(paraquat-p-phenylene) (CBPQT^4+^) ring is mechanically interlocked with a molecular axle bearing two electron-rich recognition units, namely, tetrathiafulvalene (TTF) and 1,5-dioxynaphthalene (DNP). The axle is further functionalized with a bulky 2,6-diisopropylphenyl ether group that enables its immobilization on silicon substrates through the formation of covalently attached monolayers using isocyanatopropyl linker chemistry. The CBPQT^4+^ ring carries a very short tether terminated by a thioctic acid ester for attachment to a gold-coated AFM tip.

They have pulled the macrocycle over the bulky end groups with the AFM tip. The very short linker connecting the ring to the tip did not allow them to detect the characteristic parabolic profile obtained when pulling single molecules. So, they had to rely on adhesion peaks typical of contact adhesion. They compared the adhesion forces for the molecules in their oxidized and unoxidized state, characteristic of the steric and electrostatic interactions present in the ground and oxidized states of R^4+^ (Fig. 2).

Control experiments were carried out to quantify the purely steric repulsion between the CBPQT^4+^ ring and the diisopropylphenyl ether stopper. Additional measurements were performed on R^6+^ molecules under oxidative conditions in order to evaluate the repulsive interaction arising between the CBPQT^4+^ ring and the oxidized tetrathiafulvalene unit (TTF^2+^), which drives the molecular actuation process. By comparing the force required for the ring to traverse the stopper in the absence and in the presence of the oxidizing agent Fe(ClO_4_)_3_, the authors were able to estimate the electrostatic repulsion barrier that ultimately governs the actuation mechanism.

The dethreading force was measured to be 66 pN under non-oxidizing conditions, while oxidation increased this value to 145 pN due to electrostatic repulsion. Using these force values together with distances obtained from molecular simulations—8.6 Å for the energy barrier associated with the passage of the CBPQT^4+^ ring over the bulky stopper and 13.0 Å for the displacement of the ring from the DNP recognition site to the TTF^2+^ dication—the difference in interaction energies was estimated to be 19 kcal mol^−1^. When this value was added to the theoretically calculated ground-state dethreading energy of 46 kcal mol^−1^, a total repulsive actuation energy of 65 kcal mol^−1^ was obtained. This value corresponds to the energy generated by oxidation of R^4+^ (repulsive actuation energy between CBPQT^4+^ and TTF^2+^) and represents an upper bound for the total actuation energy of the system.

However, because the experiment requires irreversible molecular rupture during the dethreading process, it is not possible to perform a relaxation step to close the actuation cycle. As a result, the mechanical work produced against an external load cannot be directly measured, and the estimated actuation energy of 65 kcal mol^-1^ cannot be experimentally validated through a complete mechanical cycle.

#### 2.1.3. Measurement of motion and intercomponent interactions in a rotaxane

**Fig. 4:**
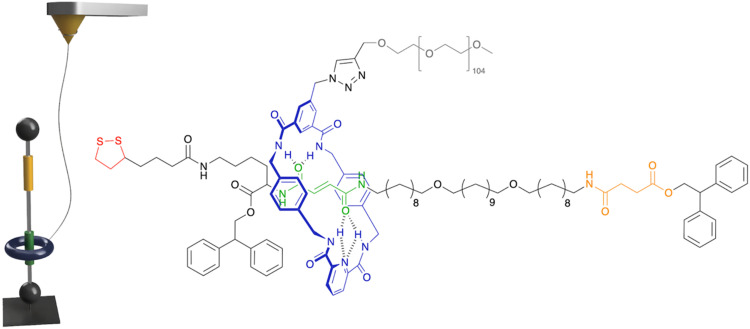
Schematic illustration of the single-molecule force spectroscopy setup applied to the hydrogen-bonded rotaxane (left) and molecular structure of the corresponding rotaxane (right). The rotaxane is immobilized on a gold surface, while the PEO tether is caught by the AFM tip. The molecular architecture comprises a benzylic amide macrocycle (shown in blue) mechanically interlocked with a linear axle terminated by bulky diphenylethyl ester stopper groups. Two recognition sites are present along the axle: a fumaramide unit (green) and a succinic amide–ester moiety (orange), both capable of interacting with the ring through up to four hydrogen bonds. Owing to its significantly stronger binding affinity, the fumaramide station is predominantly occupied by the macrocycle, with the estimated occupancy ratio exceeding 95 : 5 relative to the succinic amide–ester site. A dithiolane functionality (red) positioned adjacent to the fumaramide unit enables anchoring of the rotaxane to gold substrates. The PEO tether (grey), attached to the macrocycle, provides a mechanical handle for coupling the molecule to the AFM probe and for monitoring ring translation along the axle.

Duwez, Leigh, and co-workers demonstrated the first quantitative measurement of the work done by a single synthetic molecular machine. They were able to resolve motions occurring at the submolecular scale within a hydrogen-bonded rotaxane and to directly quantify the mechanical work generated by this system under an applied load.^30^ To achieve this, a hydrogen-bonded rotaxane was designed in which the ring component was functionalized with a comparatively long poly(ethylene oxide) (PEO) tether, enabling its displacement to be monitored using an AFM tip (Fig. 4). The resulting [2]rotaxane architecture comprises a macrocycle mechanically interlocked with a linear axle, whose ends are capped with bulky stopper groups that prevent dissociation of the assembly. The thread bears a fumaramide (green in Fig. 4) and a succinic amide-ester site (orange in Fig. 4), each of which can bind to the macrocycle through hydrogen bonds. The occupancy ratio of the fumaramide site by the ring is higher than 95%, compared to less than 5% for the succinimide ester. Adjacent to the fumaramide recognition site, a 1,2-dithiolane moiety was incorporated to allow immobilization of the rotaxane on a gold substrate *via* the formation of gold–sulphur bonds.

In these experiments, the AFM tip was employed to grab the tethered chain, thereby exerting a controlled mechanical force on the rotaxane ring and monitoring its displacement along the axle. Captured molecules were stretched in a reproducible manner by retracting the tip from the surface at a constant velocity, while the corresponding force–extension curves were recorded. Owing to the presence of the long tether, the release of the ring from its recognition site could be directly identified in the stretching traces. At low extensions, gradual elongation of the PEO chain produced the characteristic parabolic response of an entropic random coil, in which loss of conformational entropy generates a restoring force. When the applied force surpassed the strength of the four hydrogen bonds stabilizing the ring at the fumaramide station, bond rupture occurred, manifested as a distinct, albeit small, peak superimposed on the main force curve. This rupture event was observed at approximately 27 pN in dimethylformamide and around 45 pN in 1,1,2,2-tetrachloroethane for a loading rate of 500 pN s^−1^, consistent with the different hydrogen-bonding capabilities of the two solvents.

The experimental force–extension data were analyzed using the worm-like chain model, an entropic elasticity model describing the relationship between polymer extension and restoring force. By fitting the curves before and after the rupture event, the increase in contour length (Δ*L*_c_) associated with disruption of the intercomponent hydrogen bonds could be extracted. The measured Δ*L*_c_ value of approximately 4 nm closely matches the distance predicted for the axle segment separating the two binding sites, thereby confirming the assignment of the rupture event to the detachment from the fumaramide binding site.

#### 2.1.4. Direct measurement of the mechanical work generated by a rotaxane

Pulling–relaxing cycles were carried out on the hydrogen-bonded [2]rotaxane (Fig. 4), during which a distinct force peak was observed and attributed to tension transmitted through the tether. This feature provides clear evidence that the macrocycle migrates from the succinic amide–ester station back to the fumaramide site and is able to generate a restoring force while rebinding in opposition to the load imposed by the AFM cantilever. In practical terms, when the ring is temporarily arrested and the force applied to the tether is slightly reduced while elongation continues, the macrocycle actively moves to reoccupy its energetically favoured binding site. These measurements demonstrate that the ring can return to the fumaramide station while working against an external force of approximately 30 pN, a magnitude of force similar to that produced by natural molecular machines, which are considerably larger systems. The mechanical work associated with this submolecular displacement was estimated to be on the order of 6 kcal mol^−1^.

#### 2.1.5. Relating the mechanical work and free energy in rotaxanes

The free energy driving the macrocycle to bind to the fumaramide binding site at zero force cannot be obtained directly from a force–distance curve. Because of dissipation, the work is not equal to the free energy. In their study, Duwez and co-workers used the Crooks fluctuation theorem to recover this energy.^30^ The mechanical work done on/by the molecules during the pulling and relaxation steps corresponds to the area under the respective force–extension curves. Analysis of the work distributions associated with rupture and rebinding events revealed a substantial overlap across a broad range of values. The two distributions intersect at a work value Δ*G* of 9.3 ± 2.3*k*_B_*T*, corresponding to 5.5 ± 1.3 kcal mol^−1^. For a loading rate of 500 pN s^−1^, the measured work closely matches the difference in binding energies between the two hydrogen-bonding motifs. This observation indicates that, under these experimental conditions, the rotaxane can convert nearly the entire energy available from hydrogen-bond formation into mechanical work directed along the applied force.

Taken together, these results demonstrate that thermally driven, biased Brownian motion within a single synthetic small molecular system can be exploited to produce directional forces. Remarkably, the magnitude of these forces approaches that generated by biological molecular machines, even though such natural systems are substantially larger.

#### 2.1.6. Dynamics in rotaxanes

More recently, the same authors monitored real-time fluctuations during pulling experiments on these rotaxanes, allowing them to identify a transient, weakly bound intermediate state involved in the shuttling process.^35^ In these measurements, the translocation of the macrocycle along the axle was mechanically induced, forcing the ring to move between the two primary recognition sites—the fumaramide and the succinic amide–ester stations. Analysis of equilibrium fluctuations revealed an additional interaction site near the center of the thread, associated with the two oxygen atoms in this region. By examining the temporal distribution of ring occupancy, the authors confirmed the existence of this intermediate binding site during shuttling in both directions.

This work provides evidence for weak hydrogen-bonding interactions that are difficult to detect using conventional techniques. It also demonstrates that subtle variations in the chemical structure of the thread can significantly affect the dynamics of ring motion, notably by slowing the transition between the two principal stations. More broadly, these findings highlight the power of single-molecule force spectroscopy as a tool for probing both the structural features and the dynamic behaviour of synthetic molecular machines.

**Fig. 5:**
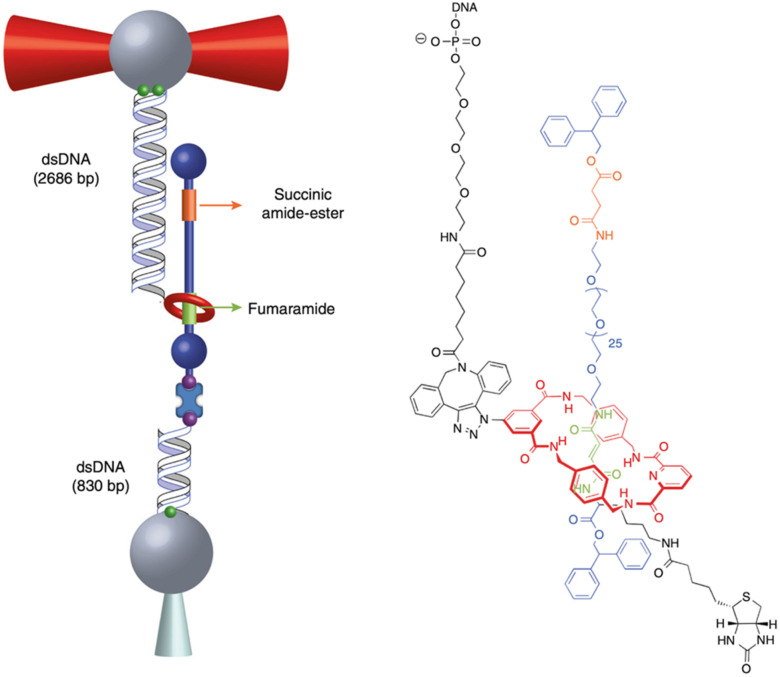
Schematic illustration of the OT-based single-molecule force spectroscopy setup applied to the hydrogen-bonded rotaxane (left) and molecular structure of the corresponding rotaxane (right). The rotaxane is attached to two polystyrene beads through two dsDNA molecules. The 2686 bp dsDNA connects the macrocycle (in red) to the bead in the optical trap through digoxigenin–antidigoxigenin (Dig–αDig) connections. The 830 bp dsDNA molecule connects the rotaxane to a bead held by suction on a micropipette, through biotin–streptavidin interactions at one end and Dig–αDig at the other end. The mechanical load is applied to the macrocycle by moving the optical trap away from the micropipette. The rotaxane consists of a benzylic tetraamide macrocycle (in red) locked onto a long axle (in blue) terminated by diphenylethyl groups at each end. The axle contains fumaramide (in green) and succinic amide-ester (in orange) stations separated by a long oligoethyleneglycol spacer. Adapted from ref. 32.

The dynamics of related H-bonded rotaxanes was also studied by Ibarra, Perez and coworkers using optical tweezers (Fig. 5). Because OT have a lower resolution on distances than AFM and can hardly operate in other solvents than water, they modified the molecule so that it had a very long hydrophilic thread that allows them to perform force experiments in an aqueous environment.^32^ Force-clamp experiments performed under low applied loads were enabled by the high stability of the trapping setup, allowing the dynamics of the rotaxane to be investigated in aqueous solution. Under these conditions, rupture of the hydrogen bonds connecting the macrocycle to the fumaramide station occurred at a lower force, approximately 8.5 pN. This value is consistent with the known solvent dependence of hydrogen bonding, which is generally weaker in water than in less polar organic solvents. Transitions between the fumaramide and succinic amide–ester stations were also detected as fluctuation events. However, no intermediate binding states were observed in these measurements. The absence of such intermediates can likely be attributed to the aqueous environment, where weak hydrogen-bonding interactions between the macrocycle and the polyether segment of the thread are significantly less favourable. Consequently, the intermediate states identified in organic solvents may become negligible in water. In addition, the spatial resolution of optical tweezers makes the detection of closely spaced intermediate states particularly challenging.

Their experimental configuration enabled highly stable pulling–relaxing measurements, making it possible to monitor shuttling events in real time. Over the course of several minutes, hundreds of transitions of the macrocycle between the two recognition sites were recorded. Analysis of these trajectories provided valuable insights into the kinetics of the shuttling process and allowed reconstruction of the corresponding energy landscape under different applied forces.

In more recent work, the authors investigated individual transition pathways of these rotaxanes under conditions of mechanical equilibrium. Their measurements revealed a broad distribution of transition-path durations, suggesting that barrier-crossing events occur independently and exhibit significant stochastic variability. Analysis of the shuttling dynamics further showed that the transition-path times were symmetrically distributed. This symmetry was corroborated by comparing the last-touch first-touch (LTFT) times measured for shuttling events in both directions.^51^

#### 2.1.7. Mechanical work and dynamics of oligorotaxanes

**Fig. 6:**
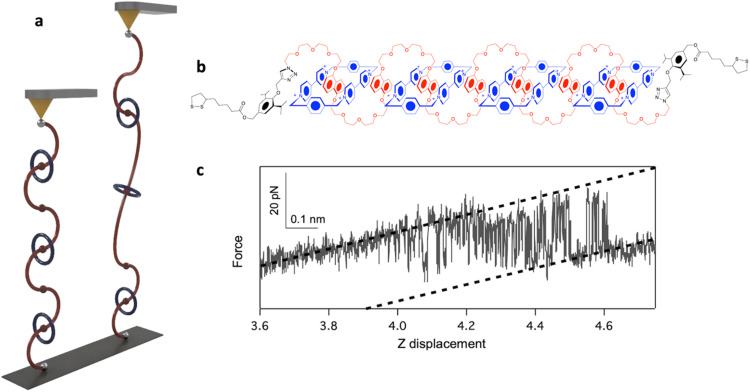
(a) Schematics of the single-molecule force spectroscopy experiment on the donor–acceptor oligorotaxane foldamer. (b) Chemical structure of the oligorotaxane, consisting of oligomeric dumbbells containing 1,5-dioxynaphthalene units threaded through cyclobis(paraquat-*p*-phenylene) rings. (c) High-resolution force–displacement curve for a single interaction within an individual [5]rotaxane molecule measured in DMF, showing clearly the hopping of the ring between distinct co-conformations. Adapted from ref. 33.

Oligorotaxanes are a class of molecules incorporating mechanical bonds in a folded molecular architecture. Donor–acceptor oligorotaxane foldamers were synthesized in 2011 by Stoddart and co-workers.^52–54^ The molecules are made of oligomeric backbone components incorporating 1,5-dioxynaphthalene units encircled by tetracationic cyclobis(paraquat-p-phenylene) rings. These oligorotaxanes were shown to adopt a robust serpentine-like folded conformation (Fig. 4), stabilized by intramolecular noncovalent interactions, including strong donor–acceptor π interactions and hydrogen bonding between the electron-rich DNP recognition sites and the electron-deficient 4,4′-bipyridinium dication units of the CBPQT^4+^. In 2018, Duwez, Sluysmans, Stoddart, and co-workers investigated the mechanochemical properties of these molecules.^33^ They used AFM-based single-molecule force spectroscopy to mechanically unfold individual molecules in dimethylformamide (DMF) (Fig. 6). A characteristic and highly reproducible sawtooth-like pattern with regularly spaced peaks was observed in the force–extension curves. The profile consisted of an alternating sequence of larger and smaller peaks, which was attributed to the stepwise disruption of intramolecular interactions between each CBPQT^4+^ ring and the neighbouring unoccupied DNP recognition sites located on either side. This alternation between strong and weaker rupture events was interpreted as a manifestation of unfolding cooperativity: once one interaction is disrupted, the corresponding interaction on the opposite side of the CBPQT^4+^ ring becomes destabilized, making its subsequent rupture easier. The rupture peaks were separated by a contour length increment of about 1.2 nm, in good agreement with the expected difference in length between locally folded and unfolded states, based on crystallographic data. The mechanical disruption of the donor–acceptor interactions stabilizing the folded oligorotaxane structure was investigated across a wide range of loading rates.^34^ The measurements revealed a consistently high rupture force over a loading rate spanning three orders of magnitude. Compared with dynamic force spectroscopy studies of various (bio)molecules conducted over the past two decades, the near-equilibrium behaviour of these oligorotaxanes persists at much higher loading rates, where biomolecules typically enter their kinetic regime. This highlights the exceptionally fast dynamics and strong rebinding capacity of the intramolecular donor–acceptor interactions. Real-time monitoring of fluctuations between folded and unfolded states demonstrated that the molecules can generate forces up to 50 pN against a mechanical load of 150 pN and exhibit transition times shorter than 10 µs.^33^ These observations indicate that folding occurs at rates at least comparable to those of proteins, while displaying notably greater mechanical robustness.

This exceptional mechanical robustness can be attributed to the presence of mechanically interlocked components, which limit the degrees of freedom and maintain close proximity of the interacting partners even after bond rupture. The interlocked architecture constrains the CBPQT^4+^ rings around the DNP recognition sites, promoting a well-defined geometry that pre-orients the binding units. By reducing the conformational freedom, this arrangement facilitates rapid refolding. Furthermore, the close spatial arrangement minimizes the entropic cost, allowing the interactions to reform quickly, even under substantial external loads, despite the small associated increase in entropy.

Single interactions at a time were probed in pulling–relaxing experiments. The observations revealed the inherently stochastic nature of the system.^34^ Even when pulling experiments are conducted close to thermodynamic equilibrium, small molecules are continuously subjected to random thermal fluctuations, which constantly influence their unfolding and refolding pathways. By applying the Crooks fluctuation theorem, the mechanical work associated with bond rupture and reformation was quantified, yielding a free-energy difference (Δ*G*) of approximately 6 kcal mol^−1^ between the two local conformations surrounding a single bond.

### 2.2. Forces in catenanes

**Fig. 7:**
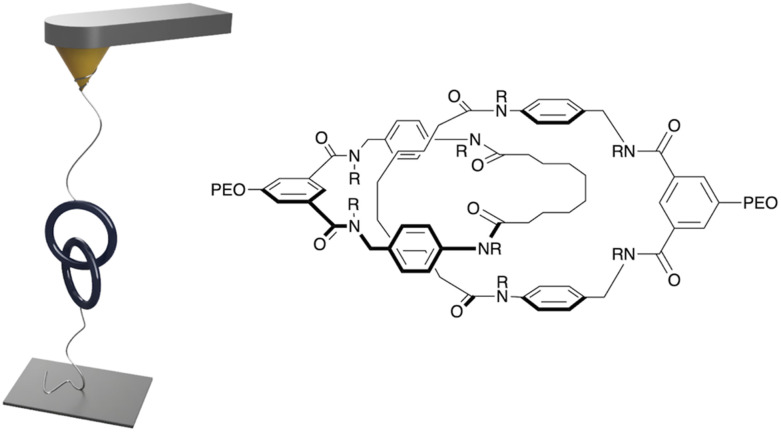
Left: Schematic representation of the single-molecule force spectroscopy experiment applied to the polymer containing the [2]catenane. Right: Chemical structure of the benzylic amide [2]catenane (CatNH: R = H and CatNMe: R = CH_3_), showing the two interlocked macrocycles and their ability to form intercomponent hydrogen bonds that modulate mobility. When R = H, H-bonds form between the two macrocycles, blocking their rotation.

In 2014, Duwez, Fustin, Leigh, and colleagues investigated the mobility of the macrocycles in benzylic amide [2]catenanes.^31^ They focused on two catenanes that differ only in the methylation of their amide groups, which directly affects their capacity to form intercomponent hydrogen bonds (Fig. 7). In the case of CatNH, the presence of secondary amide groups enables hydrogen bonding between the two macrocycles, thereby restricting their relative motion, forcing the catenane to adopt a ‘locked’ conformation. In catenane CatNMe, the amide groups are methylated, unlocking the two macrocycles so that they can freely rotate. Poly(ethylene oxide) chains were grafted on each outer edge of the [2]catenane (Fig. 7) for pulling with the AFM tip. Standard pulling experiments were carried out on the catenanes adsorbed on a substrate in both TCE and DMF. The apparent persistence length of each molecule was determined by fitting the force–extension curves using the worm-like chain model. For CatNMe, which cannot form inter-macrocycle hydrogen bonds, the persistence length (*L*_p_) was identical in both solvents (0.45 nm). In contrast, CatNH exhibited a marked increase in *L*_p_ from 0.5 nm in DMF to 1 nm in TCE, reflecting the formation of strong hydrogen bonds between the two macrocycles, effectively converting the catenane into a longer, more rigid segment. These findings demonstrate that single-molecule force spectroscopy can directly probe the mobility of macrocycles within a single catenane and elegantly illustrate the profound influence that a single mechanical bond can exert on the mechanical properties of polymeric systems.

**Fig. 8:**
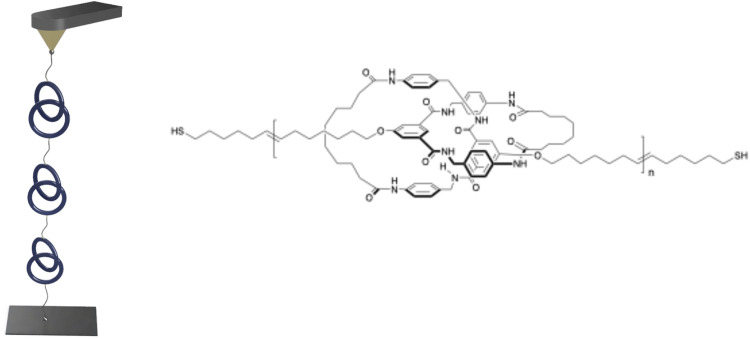
Left: Schematic representation of the AFM-based single-molecule force spectroscopy experiment on a poly[2]catenane. Right: Chemical structure of the poly[2]catenane.

Later, Zhang, Huang, and colleagues studied a poly[2]catenane composed of repeating benzylic amide [2]catenane units and separated with short alkane oligomers (Fig. 8).^55^ Similar to the CatNH in the studies by Fustin and Duwez *et al.*, these catenanes are able to form hydrogen bonds between the macrocycles, forcing the polycatenane to adopt a ‘locked’ conformation. Upon stretching, the breaking of the hydrogen bonds within the catenanes occurs, allowing the rings to slide on each other. The resulting force–extension curves show a regular sawtooth pattern containing evenly spaced peaks, with a contour length increment consistent with the expected change in length (1.5 nm) for the rupture of the inter-component hydrogen bonds obtained from the theoretical modelling of the structures. The authors also carried out dynamic experiments to study the rate of the 'unlocking' process. Upon dynamic pulling, they measured a constant increase of the rupture force with increasing loading rate, indicating that the system is driven out of its equilibrium. Using a Bell–Evans model^56^ they estimated the rate of spontaneous unlocking at about 0.07 s^−1^. Because these experiments probed the near-equilibrium regime, it would be valuable to reanalyze the data using the Friddle–Noy–De Yoreo model, which accounts for both the near-equilibrium regime—where the force is largely independent of the loading rate—and the kinetic regime, in which force scales with the logarithm of the loading rate, providing a more accurate estimation of molecular parameters.^57^ Pulling–relaxation cycles were also performed, demonstrating that hydrogen bonds between the macrocycles of the catenanes can reform during the relaxation step. By measuring the rebinding force at different loading rates, the spontaneous rebinding rate was estimated to be 3.6 × 10^5^ s^−1^ using the Bell–Evans formalism. The large difference between the unlocking and relocking rates shows the high affinity of the rings due to strong hydrogen bonding.

**Fig. 9:**
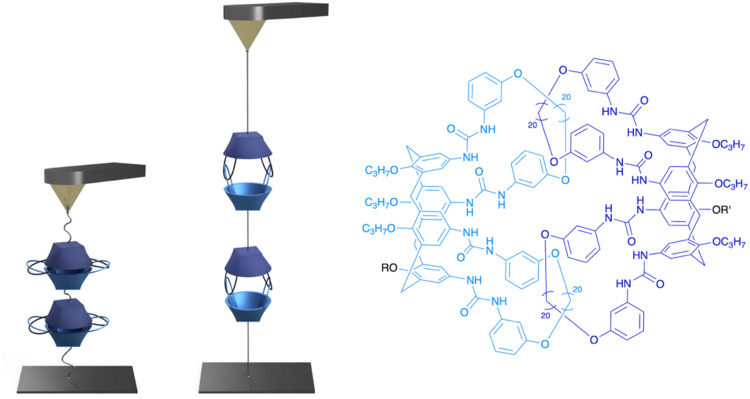
Left: Schematic representation of the AFM-based single-molecule force spectroscopy experiment applied to interlocked calix[4]arene dimeric capsules. Right: Chemical structure of the dimeric capsules, showing the 16 hydrogen bonds that hold the two units together, providing both stability and reversible binding for mechanical probing. R = C_10_H_20_CO– and R′ = C_10_H_20_NH–.

Another study of the hydrogen bond dynamics in interlocked structures was carried out by Janshoff, Marszalek and co-workers on an oligomeric system made of calix[4]arene dimers (Fig. 9).^58^ The dimers are held together by 16 hydrogen bonds, divided into 8 strong and 8 weak bonds due to different N⋯O distances. The molecular design ensures that bond rupture remains reversible, allowing the rebinding of the calix[4]arene dimers to be studied. The pulling curves showed a regular sawtooth pattern indicative of the successive rupture of the calixarene dimers. The distribution of the subsequent length increase measured in the pulling experiments revealed an intermediate state during the extension, as predicted by simulations. The hysteresis obtained during the relaxing phase at low loading rates indicates that the system is driven far from equilibrium, complicating the rebinding of the bonds. Using stochastic modelling with a three-well potential under external force, the experimental data were used to reconstruct the system's energy landscape.

**Fig. 10:**
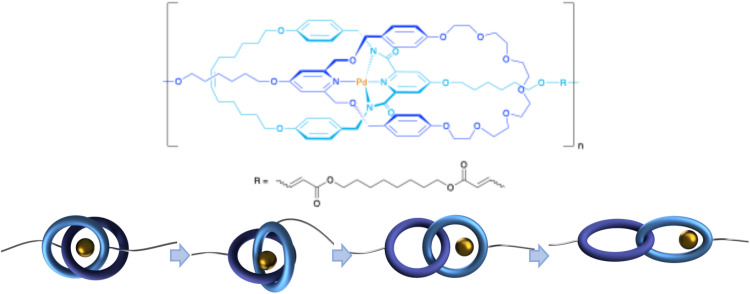
Chemical structure of the oligo[2]catenane made of palladium coordination-templated [2]catenanes, in which the two interlocked macrocycles are connected to the linkers *via* their inner edges (upper panel) and representation of the movements of the catenanes: circumrotation, translation, and elongation under the external force (lower panel).

More recently, Yan and Cao designed an oligo[2]catenane constructed from palladium-coordination-templated [2]catenanes, in which the two interlocked macrocycles are attached to the linkers through their inner edges, specifically the hydroxyl-functionalized pyridyl rings that participate in metal coordination (Fig. 10).^59^ The introduction of a strong coordination bond stabilizes the conformation of the [2]catenane and also enables the dissipation of energy through its dissociation upon applying force. In addition, force-bearing points on the inner edges of the catenane units further maximize the energy dissipation *via* the subsequent intramolecular motions of circumrotation, translation, and elongation under the external force. The force-bearing points on the [2]catenane ensure that the intramolecular motions are at their maximum. The force-extension curve exhibited a regular sawtooth pattern containing evenly spaced peaks (Δ*L* of about 7.3 nm). Such a pattern was attributed to the sequential rupture of the Pd–N coordination bonds in the [2]catenane macrocycles, in agreement with theoretical simulations, where upon stretching, the size change of a repeating monomer of the oligo[2]-catenane was found to be equal to 7.1 nm. The mean rupture force extracted from the force curves peaks at 588 ± 233 pN, which the authors attribute to the rupture of the N–Pd coordination bond. However, this assignment may be difficult to reconcile with the broad force distribution observed (standard deviation exceeding 200 pN), which suggests that multiple rupture processes may contribute to the measured signal. In particular, it appears that events associated with larger rupture forces are accompanied by a doubled revealed length, a feature that does not seem to have been explicitly considered in the analysis. This observation points toward the presence of a bimodal force distribution: one population centred around ∼400 pN, associated with a revealed length of approximately 7 nm, consistent with the expected extension for the opening of a single monomer unit, and a second population between 600 and 800 pN, corresponding to a revealed length of about 14 nm, which may arise from the simultaneous rupture of two supramolecular interactions. In addition, it is important to consider that the measured rupture force may not originate solely from the Pd–N coordination bond itself, but may also reflect the contribution of other interactions that stabilize the catenane in its “locked” conformation. These may include secondary noncovalent interactions and geometric constraints imposed by the interlocked architecture, which can significantly influence the force required to induce structural rearrangement. As a result, the measured force should be interpreted as an effective rupture force for the overall interaction network, centred around the multidentate Pd–N coordination motif, rather than as a direct measure of an isolated Pd–N bond strength. This interpretation is further supported by previous SMFS measurements on related pyridine ligand-based Pd–N coordination complexes measured in the same solvent,^60^ in which rupture forces in the range of 50–200 pN were reported, significantly lower than the values observed here.

In this context, a detailed COGEF analysis performed in the solvent used for SMFS would be highly valuable to further identify the key interactions involved and to clarify their respective roles in the mechanical response, in particular, by elucidating how force is distributed within the multidentate coordination environment. Combined with a refined analysis of the force–extension curves, taking into account the characteristic signatures discussed above, this could enable a more reliable assignment of the rupture events and help disentangle single- from multiple-bond rupture processes. Such an approach would ultimately allow for a more accurate determination of the intrinsic strength of an individual supramolecular interaction. Nevertheless, the interaction remains very strong overall, and there is no doubt that the catenane system exhibits a high degree of mechanical robustness.

### 2.3. Forces in a trefoil knot

**Fig. 11:**
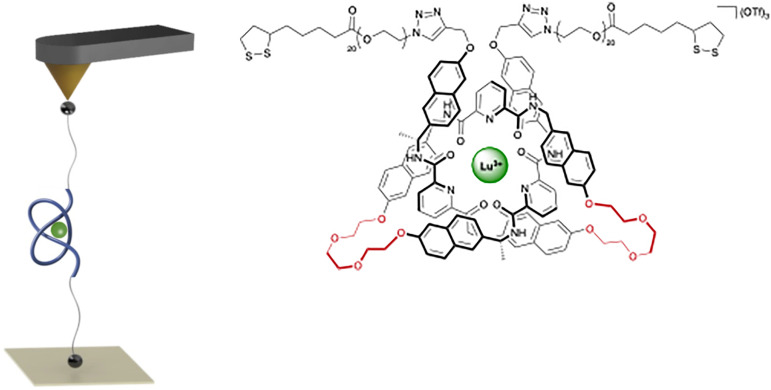
Left: Schematic representation of the AFM-based single-molecule force spectroscopy experiment used to mechanically tighten a trefoil knot. The knotted conformation is stabilized in solution through coordination with a Lu^3+^ ion (green). Two functionalized PEG chains serve as tethers, enabling the knot to be captured between the AFM tip and the substrate. Right: Chemical structure of the trefoil knot.

In 2023, AFM-based SMFS combined with quantum chemical (QC) calculations was used to investigate the mechanical properties of a synthetic small-molecule trefoil knot (Fig. 11).^61^ The system consists of a single strand bearing three tridentate 2,6-pyridinebiscarboxamide motifs, which fold around a nine-coordinate Lu^3+^ ion to form an open trefoil (overhand) knot. Note that in this open molecular knot, unlike a closed knot, the topology is only preserved by the presence of the metal ion template and unravels when the template is removed. The authors analysed how the knot responds mechanically upon tightening in different solvents, measuring the force needed and contrasting it with the behaviour of a corresponding unknotted strand. They showed that the force is modulated by the solvent—on the order of 100 pN in 1,1,2,2-tetrachloroethane and 60 pN in acetonitrile—in agreement with the internal coordination interactions. They estimated the extra energy that the knot can accommodate in comparison with a corresponding unknotted strand. Indeed, the presence of a characteristic deviation from a WLC behaviour was shown to be characteristic of an increase in the extensibility of the molecule. The additional mechanical energy that a single molecular strand can withstand under an external load due to the presence of a knot along the chain was estimated to be 13 kcal mol^−1^ in 1,1,2,2-tetrachloroethane. Pulling–relaxing experiments further indicated that the metal ion remains coordinated within the knotted structure during the tightening process. This persistent coordination facilitates the rapid and efficient recovery of the initial knotted conformation, with little to no hysteresis observed. These findings highlight the relatively high rigidity of such synthetic small-molecule knots and demonstrate their greater resistance to externally applied forces compared with naturally occurring biological knots.

### 2.4. Key insights and outlook from single-molecule force spectroscopy

Taken together, these studies demonstrate the remarkable potential of SMFS to probe MIMs at an unprecedented level of detail. Beyond the simple measurement of rupture forces, SMFS enables direct access to fundamental properties, including the strength of noncovalent interactions, intramolecular motion, mechanical work, and energy landscapes governing molecular dynamics. In rotaxanes, it has been possible to quantify submolecular displacements, measure the mechanical work generated by shuttling processes, and relate force-induced transformations to thermodynamic quantities through fluctuation theorems. In more complex architectures such as oligorotaxanes, catenanes and knots, SMFS has revealed cooperative effects, fast rebinding dynamics, and the key role of mechanical bonds in enhancing structural robustness and enabling efficient energy dissipation.

Furthermore, these approaches provide unique insights into dynamic processes, such as stochastic shuttling, intermediate binding states, and transition pathways, which are often inaccessible by ensemble techniques. The comparison between different experimental platforms, such as AFM and optical tweezers, also highlights the strong influence of the solvent environment, timescales, as well as spatial and temporal resolution on the observed behaviour.

Despite these advances, several challenges remain. The interpretation of force spectroscopy data can be complex, particularly when multiple rupture pathways or overlapping processes contribute to the measured signals. Achieving precise control over molecular attachment, improving temporal and spatial resolution, and developing robust analytical frameworks to disentangle single- from multi-event processes are key areas for future progress. In this context, combining SMFS with complementary theoretical models and simulations will be essential to fully exploit its potential and to achieve a quantitative understanding of force transmission mechanisms and the conversion of chemical energy into mechanical work at the molecular scale.

## 3. Polymer mechanochemistry with mechanically interlocked molecules

In polymer mechanochemistry, force is applied through polymer chains to force-responsive sites called mechanophores.^62^ These mechanophores can have multiple responses to applied force including damage detection,^63–66^ catalysis,^67,68^ material strengthening,^69^ production of challenging macromolecular structures,^23,24,70,71^ the unlocking of unusual reaction pathways,^72–75^ the generation of reactive sites,^76–78^ or even the release of small molecules,^79–85^ In the solid state, force can be applied by the compression or stretching of materials. High levels of force (nN regime) can be achieved in solution using ultrasound-induced cavitation, as elongation flows are created in the vicinity of collapsing cavitation bubbles. Any polymer chain, which is caught in the flow, will become rapidly extended until covalent bond scission occurs, usually in the central region of the chain.^86^

Mechanical bonds in the centre of a polymer chain will therefore undergo forces high enough to cleave covalent bonds and at a much higher strain rate than is achieved using SMFS, allowing for the study of the force-induced dissociation of the mechanical bond.^87^ The study of force-promoted covalent bond cleavage within mechanical bonds has become increasingly important with the development of mechanically interlocked materials, which have the ability to absorb mechanical energy at low levels of deformation (see Section 4), and devices, which can use mechanical force as a stimulus to elicit a useful response (see Section 3.2). Knowledge of covalent bond scission pathways at high levels of force will aid the design of future force-responsive materials and lead to the development of interlocked molecular systems with unique behaviour and applications. Similarly, the combination of a mechanical bond with a directional force offers great potential for the development of force-driven molecular machines and devices.

**Fig. 12:**
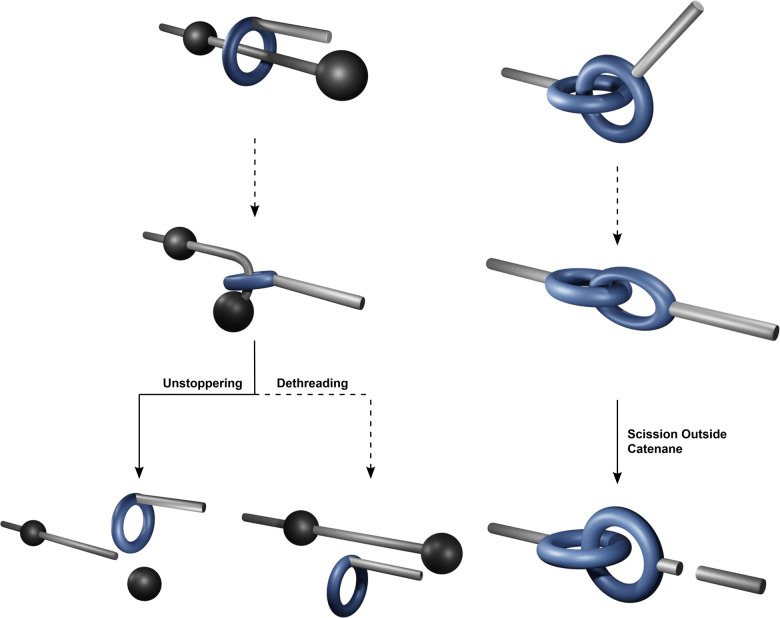
Force-promoted dissociation of mechanical bonds. Mechanical force pulls component parts towards each other, resulting in either the accelerated dissociation of the mechanical bond or cleavage outside the architecture. Dashed arrows indicate non-covalent processes. Solid arrows indicate covalent bond scission events.

Force can provide the energy for the dethreading of a thermally stable rotaxane with a small stopper.^50,88–90^ In systems with a stopper sufficiently large to prevent dethreading, or in a catenane where component parts cannot be separated without covalent bond scission, the interlocked architecture can strongly influence the cleavage of the covalent bonds which it contains (Fig. 12). This section will discuss the mechanical strength of rotaxanes, catenanes, and knots, followed by force-responsive systems that utilise the dissociation of these architectures for useful force responses.

### 3.1 Mechanochemistry of mechanical bonds

#### 3.1.1 Non-covalent dissociation

**Fig. 13:**
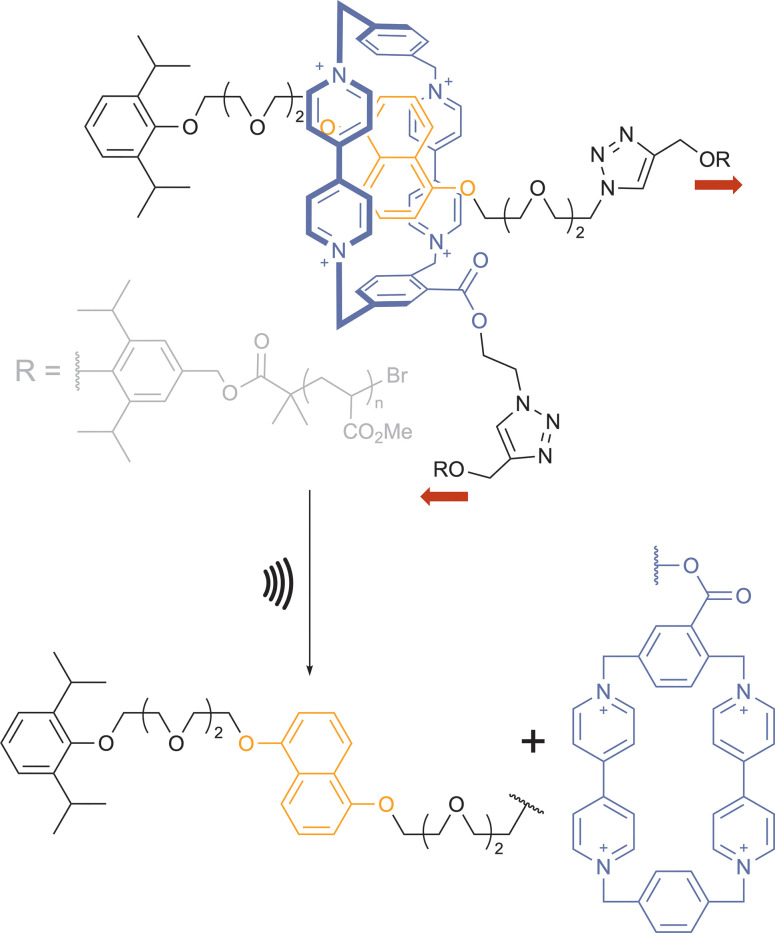
The force-promoted dissociation of Stoddart's rotaxane is likely to occur by dethreading. Conditions: US (6.0 W cm^−2^), THF, 0 °C, 60 min. Red arrows indicate the direction of force. PF_6_^−^ counter ions are omitted for clarity. The cyclobis(paraquat-*p*-phenylene) ring is shown in blue and the 1,5-dioxynaphthalene unit in orange.

The application of force to mechanical bonds has the ability to disrupt the intramolecular interactions between the component parts, allowing for the movement of the components with respect to each other.^87^ As a macrocycle is pulled down an axle, towards the stopper, mechanical force has been shown to provide the energy for the macrocycle to pass over small stoppers. As explained in detail in Section 2, an early AFM study by the Stoddart group showed that the dethreading of a cyclobis(paraquat-*p*-phenylene) macrocycle over a 1,5-dioxynaphthalene stopper was possible at 66 pN (Fig. 3).^50^ Although the AFM study provides a measurement of the level of force required to dissociate a mechanical bond, no further details can be obtained pertaining to the dissociation mechanism or resulting products. The use of ultrasonic activation in solution can allow for analysis of the species formed from application of force and give more insight into the chemistry that occurred at the mechanical bond under force. The first dissociation of a rotaxane by ultrasonication was shown by the same group in 2011 (Fig. 13).^88^ When force was applied to a cyclobis(paraquat-*p*-phenylene)-derived [2]rotaxane, *via* high intensity ultrasound in solution, the authors observed a decrease of the UV-vis absorption of the polymer due to the disruption of the charge transfer between the macrocycle and the electron rich 1,5-dioxynaphthalene unit on the axle. This decrease was greater for the rotaxane mechanophore compared to the non-interlocked axle and a force-uncoupled rotaxane positioned at the end of a polymer chain, suggesting that the force-promoted destruction of the rotaxane was the cause of this effect. AFM experiments^50^ and CoGEF (constrained geometries simulate external force) calculations^91^ indicate that the macrocycle is able to pass over the stopper of this rotaxane at forces much lower than that required for covalent bond scission,^92^ suggesting a dethreading mechanism of dissociation.

After this initial example of force-induced dethreading of a rotaxane, the same dissociation mechanism has been used in the design of force-responsive materials. For instance, a crown ether-based rotaxane has been used as a sacrificial mechanical bond to enhance the stability of cross-linked materials,^93^ while a dethreading mechanism has been used to yield an irreversible fluorescence emission in a dual response rotaxane system (see also Section 3.2.1).^89,90^

#### 3.1.2 Covalent dissociation

When dethreading is not possible (*i.e.*, when the stopper is much larger than the cavity of the macrocycle), the force build-up in the mechanical bond will pull the component parts against each other, inducing the distortion of bond angles and the elongation of covalent bonds at the contact point (intermediate structures in Fig. 12). Different types of architectures have contrasting effects on the stability of their constituent covalent bonds: from accelerated bond scission in rotaxanes and knots to the force-protecting effect of a catenane.

##### 3.1.2.1 Strength of a rotaxane

**Fig. 14:**
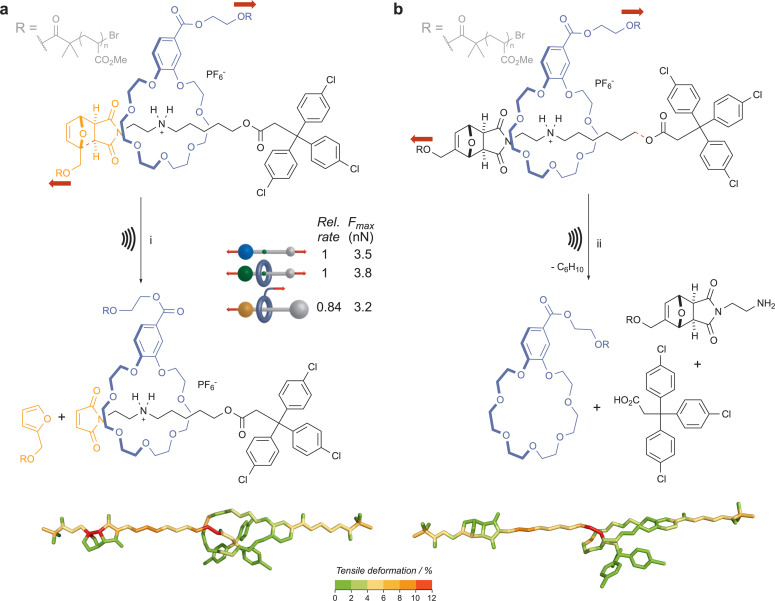
Rotaxanes influence the mechanochemical reactivity of covalent bonds in the axle. (a) A rotaxane as a force actuator in a DA reaction. Conditions: (i) US (20 kHz, 13.0 W cm^−2^, 1 s ON/2 s OFF), THF, 5–10 °C, 240 min. (b) Mechanical dissociation of a rotaxane mechanophore by an unstoppering process. (ii) US (20 kHz, 11.5 W cm^−2^, 1 s ON/2 s OFF), CH_3_CN, 5–10 °C, 240 min. Red arrows indicate the direction of force, and the first scissile bond is shown as a red dashed line. Stress distribution determined by CoGEF calculations (DFT B3LYP/6-31G*). The benzo-21-crown-7 (B21C7) macrocycle is shown in blue and the furan-maleimide DA adduct in orange.

In 2019, Zhang and De Bo showed how the rotaxane architecture affects the mechanical reactivity of covalent bonds within its axle (Fig. 14).^94^ A benzo-21-crown-7 (B21C7) macrocycle was stoppered on one side by a mechanically active Diels–Alder (DA) mechanophore^95^ and on the other side by a large stopper preventing dethreading. Application of force on the rotaxane by high intensity ultrasound pulls the macrocycle along the axle until it reaches the stopper, and further elongation increases the tension in the whole rotaxane, resulting in the cleavage of the DA adduct (observed by ^1^H NMR spectroscopy). DA adduct cleavage occurred at a lower rate in the rotaxane compared to the axle or force-uncoupled rotaxane due to the constriction of the axle as the macrocycle is pulled against the stopper. CoGEF calculations showed the development of two competing high-stress regions, at the DA adduct and the axle-stopper junction. These competing regions result in lower mechanochemical coupling to the DA adduct and a slower rate of dissociation due solely to the rotaxane architecture. If a significantly weak bond is not present in the axle, this high-stress region may instead result in accelerated cleavage of the rotaxane at the axle–stopper junction. Indeed, when the axle included a mechanically inert DA adduct,^95^ sonication resulted in the homolytic cleavage of the C–O bond at the junction (dashed red bond in Fig. 14b), with the carboxylic acid of the stopper and of the C–N bond on the right side of the ammonium station, liberating the macrocycle, the amine-terminated polymer, and the hexyl linker, as observed by ^1^H NMR spectroscopy.^96^ This behaviour is unique to the rotaxane architecture as the non-interlocked and polymer end group control structures cleaved randomly in the poly(methyl acrylate) (PMA) backbones. CoGEF calculations revealed that the constriction of the axle by the stretched macrocycle generates significant tensile, bending, and torsional stresses, which eventually trigger the cleavage of an otherwise inert covalent bond at the axle–stopper junction. It was subsequently shown that the extent of unstoppering in a rotaxane can be altered by the ability of the macrocycle to undergo internal change of conformation under the application of force, which acts as a mechanical damper (see Section 3.2.3).^97^

Taken together, these studies show how careful design of a rotaxane can be used to control the reactivity of the covalent bonds within the architecture. These force-responsive properties of rotaxanes will have important implications for the development of future materials, where fragments of the rotaxane can be released on demand, and for the design of slide-ring materials (Section 4) as this architecture may accelerate the failure of these materials at high levels of force.

##### 3.1.2.2 Strength of a knot

**Fig. 15:**
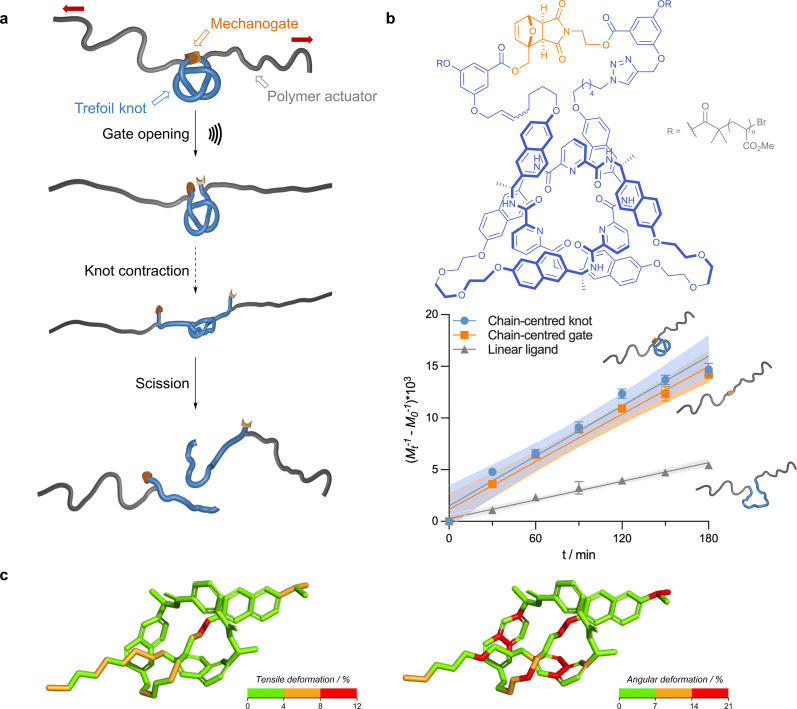
Force-promoted dissociation of an overhand knot. (a) Cartoon representation of knot dissociation. Conditions: (i) US (20 kHz, 11.5 W cm^−2^, 1 s ON/2 s OFF), CH_3_CN, 5–10 °C, 180 min. Red arrows indicate the direction of force. (b) Chemical structure of the knot and dissociation kinetics of the knot, gate and linear ligand. The potential scissile bond is shown as a red dashed line. (c) Stress distribution determined by CoGEF calculations (DFT UB3LYP/6-31G*). The knot is indicated in blue and the furan-maleimide DA adduct in orange.

Behaviour similar to the accelerated cleavage at the contact point between component parts of a rotaxane is observed in macroscopic materials, where the presence of a knot will substantially weaken the materials at the entrance to the entanglement,^98^ reducing the tensile strength of ropes. As knots form spontaneously in any polymer of sufficient length,^99^ chemists have long wondered if the same effect would be observed at the molecular level. Computer simulations^99^ and theory^100,101^ have suggested that the same behaviour will occur on the nanoscale; this proposal was verified experimentally in 2024.^102^ A polymer chain containing an overhand (*i.e.* an open trefoil, 3_1_) knot, gated with a mechanochemically-active DA mechanophore to prevent the translocation of the knot along the polymer chain, was activated by high intensity ultrasound in solution. Mechanochemical cleavage of the DA adduct exposes the open overhand knot which will tighten further, without having time to unravel, leading to covalent bond cleavage. The knotted polymer chain cleaved at a faster rate than the linear ligand, a compound with the same chemical structure as the knot but in a non-knotted form and at a similar rate to a polymer containing only the DA mechanophore (suggesting that the gate opening is the rate-limiting step of the knot scission). The linear ligand did not contain a mechanically weak site and hence bond cleavage occurred randomly in the polymer chain. In contrast, the knotted polymer was shown to cleave at the C–O bond adjacent to the naphthyl group (indicated in red in Fig. 15b), which is located at the entrance of the knot in the contracted structure (Fig. 15c). Indeed, CoGEF calculations showed that the naphthyl C–O–C bonds are distorted and the naphthyl groups themselves significantly bend before heterolytic cleavage of the naphthyl C–O bond occurs where the strand exits the loop of the knot, with the growing positive and negative charges stabilised by the pyridine lone pair and amide NH inside the knot. The knot has a lower force required for bond cleavage than even the DA mechanophore used as the gating structure, making it one of the most reactive scissile mechanophores reported.^103^ This study also provided further evidence that the behaviour of a knot under tension is solely due to the topology of the architecture and is independent of the material, with the same stress distribution and scission point occurring across length scales.^98^ With similar behaviour to the rotaxane, a molecular knot can activate an otherwise unreactive bond upon application of mechanical force due to the tension between strands in the architecture, which is another key consideration in future polymer material design.

##### 3.1.2.3 Strength of a catenane

**Fig. 16:**
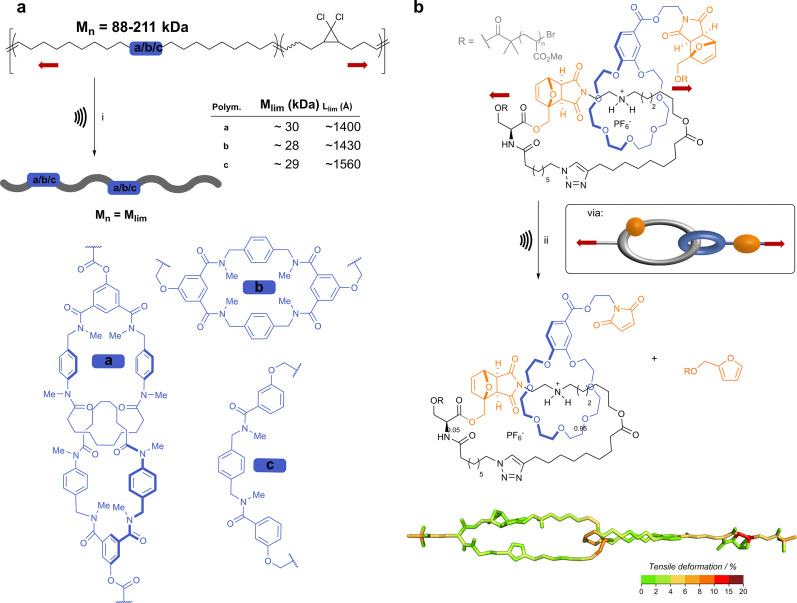
Catenanes act as mechanochemical protecting groups. (a) Sonication of multimechanophore polymers containing catenanes, macrocycles or linear sections. Conditions: (i) US (20 kHz, 8.5 W cm^−2^, 1 s ON/2 s OFF), THF, 5–10 °C, 240 min. (b) Mechanical activation of a catenane mechanophore. (ii) US (20 kHz,13.0 W cm^−2^, 1 s ON/2 s OFF), CH_3_CN, 5–10 °C, 240 min. Stress distribution determined by CoGEF calculations (DFT B3LYP/6-31G*). Red arrows indicate the direction of force, and the first scissile bond is indicated by a red dashed line.

Although the distortion of the axle of a rotaxane causes accelerated covalent bond cleavage, the macrocycle is relatively strain free. The circular backbone allows the tension to spread over the whole structure, meaning that macrocycles are less susceptible to force-induced deformation or bond cleavage. In 2016, Craig and co-workers showed that the inclusion of [2]catenane junctions has no impact on the stability of copolymers (Fig. 16a).^104^ A *gem*-dichlorocyclopropanated-polybutadiene copolymer containing 5 mol% of benzamide [2]catenanes was sonicated until the limiting molecular weight (*M*_lim_) was reached, below which no further mechanochemical reactivity can occur. The *M*_lim_ was consistent with non-interlocked or macrocyclic controls, showing that the architecture had no impact on the cleavage, which likely occurred in the linear linkers connecting the catenanes together; however, no more information on the location or nature of cleavage was available. A later study by Zhang and De Bo proved that tension is greater outside of the macrocycles of a catenane than within the macrocycles themselves by placing identical DA mechanophores inside one macrocycle of a [2]catenane and in the linear segment linking the supramolecular structure to the polymer chain (Fig. 16b).^105^ The catenane featured a large macrocycle containing a scissile proximal-*exo* DA adduct^94^ and an ammonium binding station surrounded by a B21C7 macrocycle linked to an identical DA adduct which was, in turn, linked to the actuating polymer. Elongation of the catenane causes the macrocycles to rotate around each other until the tension is spread equally across the two rings (box in Fig. 16b). This causes the tension to accumulate in the linear segments outside of the macrocycles, notably at the site of the extracyclic DA adduct (Fig. 16b). ^1^H NMR spectroscopy after sonication showed the exclusive activation of the extracyclic DA adduct, confirming that cleavage occurs outside the macrocycles of a catenane and that the architecture can be used to completely shield a reactive site from force.

The behaviour of each type of mechanical bond is specific to its architecture; rotaxanes and knots induce stress, while a catenane will diffuse it. The significant tightening of a molecular knot renders it one of the weakest covalent mechanophores reported to date. On the other hand, catenanes can completely diffuse the applied stress and will not significantly weaken the polymer chain that contains them. These findings further show how the design of mechanical bonds can allow for precise control of the reactivity of force-responsive sites within their component parts and pave the way for the development of force-controlled molecular devices.

### 3.2 Mechanochemistry using mechanical bonds

#### 3.2.1 Change in optical properties

The dissociation pathways described above have also been harnessed to access force-responsive systems which use the destruction of the mechanical bond to give a unique and specific response to force. External forces can provide the energy required to overcome intramolecular interactions between the component parts and result in a reversible observable response. If the mechanical bond can dissociate by a dethreading pathway, this response becomes irreversible, allowing the rotaxane architecture to be used to detect different levels of applied force.

**Fig. 17:**
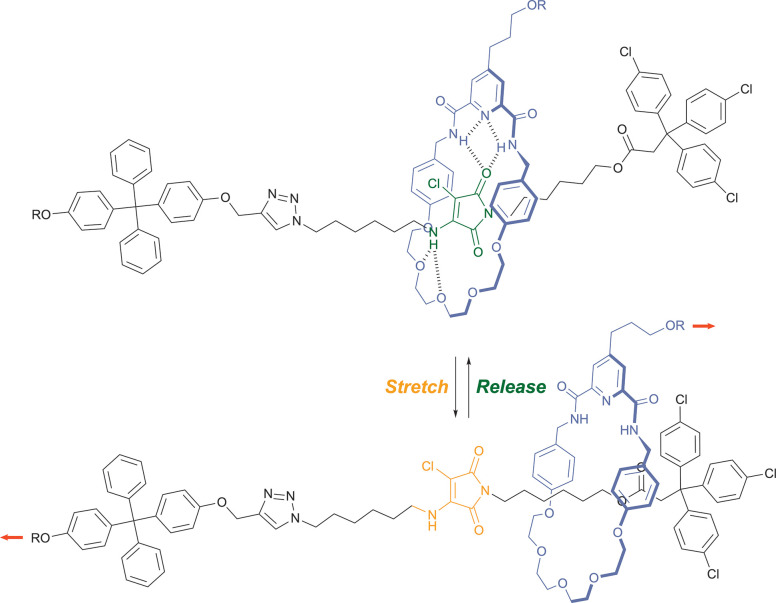
Concept of a mechanochromic hydrogen bonded rotaxane based on the O’Reilly dye. In the stretched state, the macrocycle moves away from the fluorophore, affecting its optical properties. Red arrows indicate the direction of the force.

Polymers which give an optical response to applied force are useful in applications such as force sensing and damage detection. There are many examples of mechanochromic molecules which rely on covalent bond cleavage and therefore require high activation forces.^106,107^ On the other hand, the moveable component parts of mechanical bonds can be separated on application of force by the disruption of non-covalent interactions, without the need for covalent bond scission, giving access to immediately reversible force sensors with the ability to sense lower forces relevant for the detection of mechanobiological processes in the pN regime.^108^ The De Bo group accessed a force sensor based on the disruption of hydrogen bonds^109^ between a pyridine bis-amide and a crown ether-containing macrocycle around an O’Reilly dye (Fig. 17).^110^ Application of force resulted in the decrease of the fluorescence of a polyacrylamide gel. Although the response was irreversible, it represented a proof of concept for the development of force sensors operating in a synthetic model of living tissue.

**Fig. 18:**
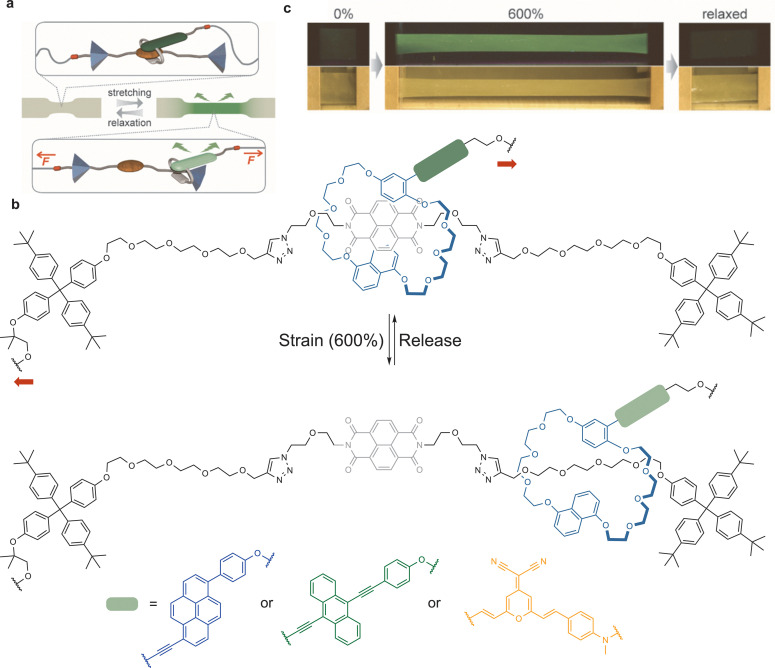
Rotaxanes as reversible force sensors. (a) Schematic illustration of a reversible rotaxane response to force. (b) Activation of rotaxane mechanophores by 600% elongation stretch and release cycles. The 1,5-dinaphtho[38]crown-10 macrocycle is shown in dark blue, and the 1,4,5,8-napthalenetetracarbocyclic diimide quencher in grey. (c) Photographs of the film before, during and after stretching. Red arrows indicate the direction of force. Images in (a) and (c) reproduced from ref. 110 with permission from the American Chemical Society, copyright 2018.

Sagara and Weder showed multiple examples of mechanochromic rotaxane systems, based on fluorophore-containing macrocycles wrapped around an electron-poor aromatic quencher (Fig. 18).^111^ Low levels of applied force are able to move a derivative of a 1,5-dinaphtho[38]crown-10 macrocycle linked to a 4,7-bis(phenylethynyl)-2,1,3-benzothiadiazole fluorophore away from a 1,4,5,8-napthalenetetracarbocyclic diimide quencher on the axle, resulting in green emission which immediately returns to the original state after the force is removed. Different colours of emission were possible by varying the luminophore, allowing access to blue (π-extended pyrene), green (π-extended anthracene), and orange (π-extended 4-(dicyanomethylene)-2-methyl-6-(4-dimethylaminostyryl)-4*H*-pyran) photoluminescence. Combining the three rotaxanes in the same elastomer gives access to white-light emission.^112^

**Fig. 19:**
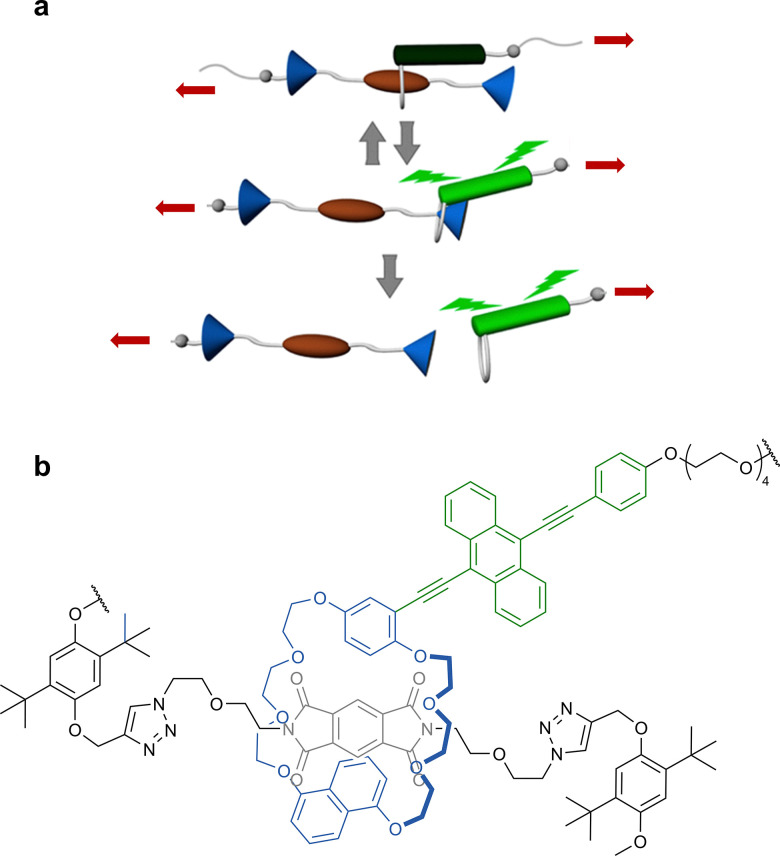
A rotaxane as a dual response force sensor. (a) Schematic illustration of a rotaxane showing both reversible and irreversible responses to force. Red arrows indicate the direction of force. (b) Example structure of dual response rotaxane. The 1,5-dinaphtho[32]crown-8 macrocycle is shown in dark blue, the pyromellitic diimide quencher in grey, and the π-extended anthracene fluorophore in green. Image reproduced from ref. 89 with permission from the American Chemical Society, copyright 2021.

In the above system, the macrocycle cannot pass over the bulky stopper, leading to reversible activation at low levels of force. Irreversible emission would be useful for yielding information on different levels of applied force and allow the determination of the stress history of a material. The same group later designed a rotaxane that can show both reversible and irreversible emission responses using an intermediate-sized stopper (Fig. 19).^89^ The design consisted of a smaller stopper (*p*-di-*tert*-butylphenyl) and quencher (pyromellitic diimide (PDMI)), and a modified 1,5-dinaphtho[32]crown-8 macrocycle. At low levels of force, the macrocycle can be reversibly pulled away from the quencher and retained by a smaller stopper. The stopper is small enough that the macrocycle can dethread above a certain force threshold, leading to an irreversible response. The same reversible response as shown above was possible for up to 50 strain/release cycles at 300% elongation; however, dethreading was possible above 600% elongation. Further studies on these rotaxane systems showed that the length of the axle had an impact on the force-response behaviour.^113^ As the ethylene glycol linker between the quencher and the stopper was lengthened, the emission intensity remains higher for longer after the strain is released. This behaviour is specific to rotaxanes in solid polymers and was not observed in solution.^114–116^ This finding allows for the axle length to be tuned to yield a material with either an immediate or delayed reversible response to force. The effect of the stopper size on dethreading was also further investigated.^90^ Similar PDMI-based rotaxanes were stoppered on the one side by a diphenylmethane-based stopper, featuring one *tert*-butyl and one 1-hydroxy-2-methylpropyl group, and on the other by either bis(4-*tert*-butylphenyl)methane, bis(4-methylphenyl)methane, diphenylmethane, 1,4-di-*tert*-pentylbenzene, or 1,4-di-*tert*-butylbenzene. Dethreading over the diphenylmethane-based stopper occurred slowly at room temperature in solution and was promoted during the solvent casting process to prepare thin films of the material. The difference between the relaxed and stretched state emission disappeared after only a few cycles, allowing this rotaxane to detect extremely low forces. No dethreading occurred after 50 stretching cycles and fully reversible mechanophore behaviour was observed for the bis(4-*tert*-butylphenyl)methane and bis(4-methylphenyl)methane stoppers. Rotaxanes with 1,4-di-*tert*-pentylbenzene and 1,4-di-*tert*-butylbenzene stoppers showed intermediate behaviours, with the strain-free emission intensity increasing with each stretching cycle allowing these rotaxanes to display a dual response. The design changes in the above studies highlight how small changes to the rotaxane architecture can result in dramatic changes in the force-response behaviour and material usefulness for different levels of stress detection.

#### 3.2.2 Force-controlled release

The disassembly of a rotaxane by covalent bond scission allows for fragments of the mechanical bond to be released. The release of small molecules offers great potential in the development of responsive materials with damage-reporting or self-healing properties and for drug delivery.^81,82,117^ Mechanical bonds offer unique small molecule-release mechanisms with the ability to release multiple complex molecules in a single activation event.

**Fig. 20:**
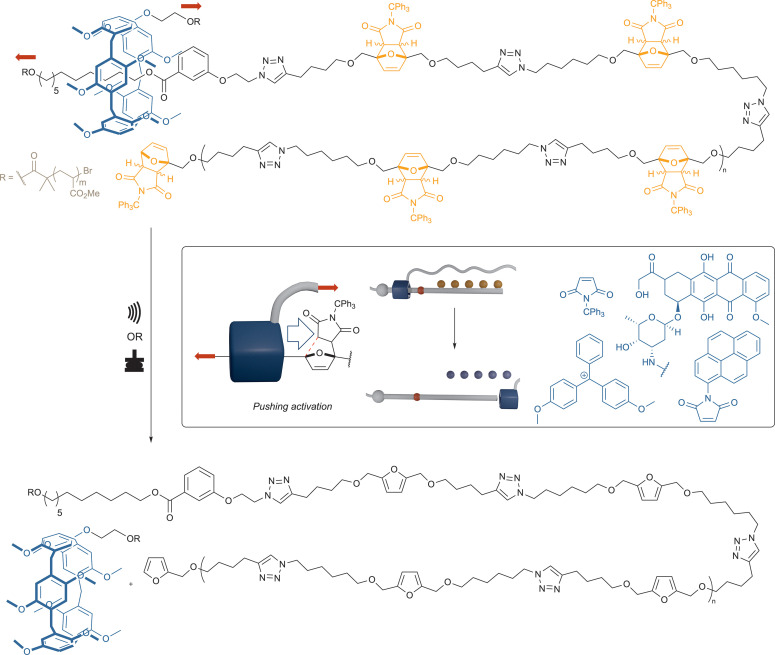
Force-promoted reactivity of a multi-cargo-release rotaxane. Conditions: (i) US (20 kHz, 13 W cm^−2^, 1 s ON/1 s OFF), CH_3_CN, 5–10 °C, 300 min. (ii) Bulk compression (0.74 GPa, less than 60 min per cycle, 10–45 cycles). Red arrows indicate the direction of force, and the first scissile bond is shown as a red dashed line in the inset.

In 2024, the De Bo group used the actuating behaviour of a rotaxane to release multiple cargo units from a single axle, based on force-promoted retro-DA reactions, as the macrocycle pushes against a sequence of up to five stoppers along the axle (Fig. 20).^118^ A pillar[5]arene macrocycle threaded around a C12 alkyl chain was stoppered on the one side by a cargo-bearing oligomer and was linked to a PMA polymer on the other. *N*-Triphenylmethyl maleimide was installed as the cargo unit *via* a DA reaction with multiple furan sites on the oligomer. As the macrocycle is pulled along the axle, each DA adduct it reaches sequentially acts as a stopper; further pulling will trigger a retro-DA reaction (*via* a pushing activation, see below) releasing the cargo unit and subsequently allowing the macrocycle to continue being pulled along the axle. The concept was first investigated in a rotaxane bearing a single cargo unit, where both *endo*- and *exo*-DA adducts were activated with a 71% efficiency to release their cargo molecule. A variety of functional molecules including a drug (doxorubicin), a fluorescent tag (*N*-(1-pyrenyl)maleimide) and an organocatalyst (trityl cation) could be released in this way, highlighting the usefulness of this system for biomedical and materials applications. The retro-DA activation could be repeated multiple times in a single activation event until the macrocycle reaches the end of the cargo-bearing oligomer or competing bond scission dissociates the mechanical bond. Rotaxanes loaded with 3 and 5 cargo molecules were activated by ultrasonication in solution, leading to cargo release of up to 44% and 22%, respectively. The major competing pathway was unselective scission in the polymer chains (up to 40% and 50% for the 3 and 5 cargo-bearing structures, respectively). Interestingly, no rotaxanes were isolated with a partial load, suggesting that the actuation releases all or none of the cargo load (the latter case being the consequence of unselective scission in the polymer chains). Activation was also shown to be possible in bulk by compression of the polymer material. In this case, release of the cargo was observed to a lower extent than in solution due to the ability of the network to distribute tension and the possibility of the rotaxane to be located in a low stress region. However, the activation efficiency of 30% for a single cargo places this work among the best covalent force-controlled release systems described to date.^81^

**Fig. 21:**
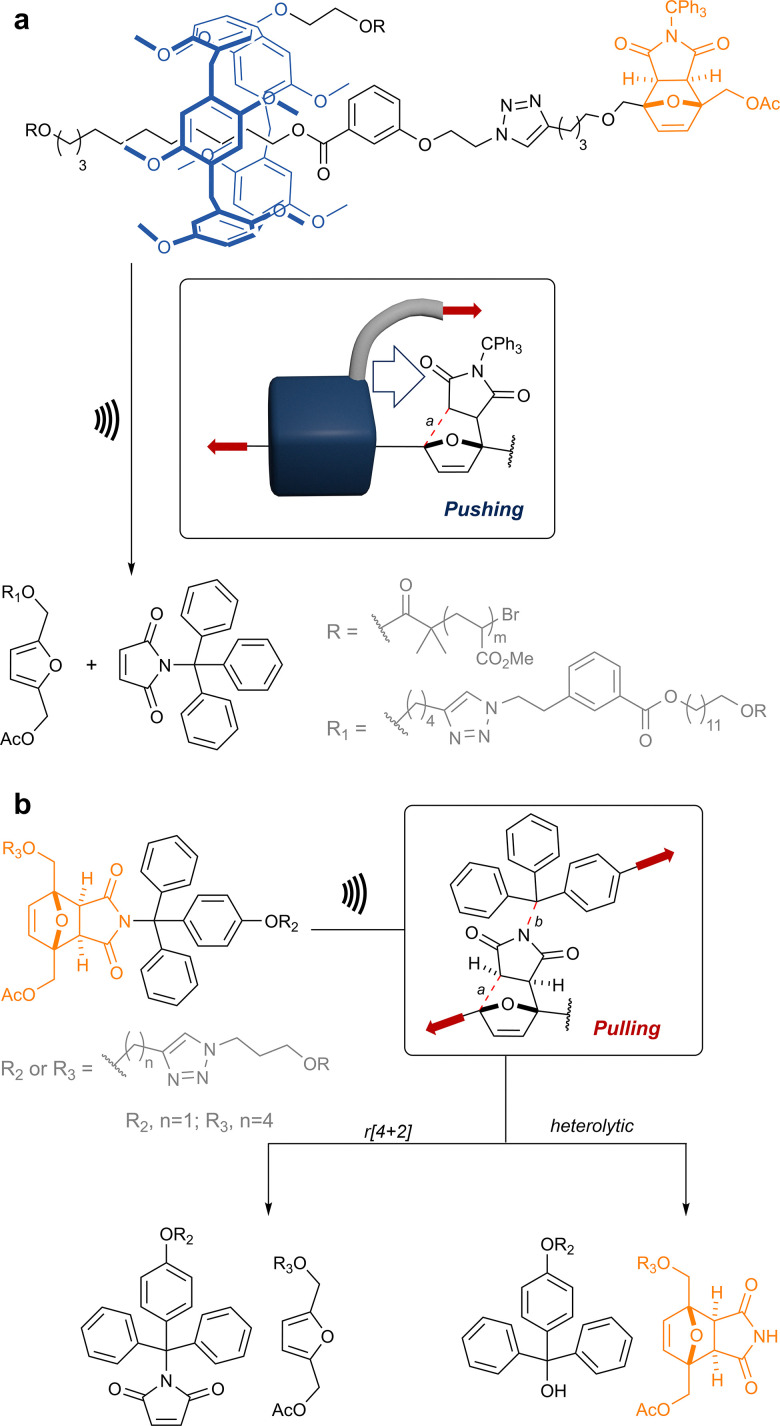
Pushing *vs.* pulling actuation for the activation of a Diels–Alder mechanophore. (a) Pushing actuation with a rotaxane actuator and (b) pulling actuation with covalently attached polymer actuators. Conditions: US (20 kHz, 13.0 W cm^−2^, 1 s ON/1 s OFF), THF/H_2_O: 75/1, 5–10 °C, 90 min. Red arrows indicate the direction of force, and scissile bonds are shown as red dashed lines.

The unique pushing activation of this rotaxane actuator was further investigated by comparing the rotaxane actuation with the traditional pulling activation obtained by covalently attaching polymers to the mechanophore (Fig. 21).^119^ Although the macromolecules are stretched in both situations, the mechanophore experiences different modes of activation, either by pushing or by pulling. In the rotaxane system, the macrocycle exerts a compressive force that effectively pushes on the mechanophore, whereas in the pulling configuration, the mechanophore is activated by tensile forces applied through the two polymer arms. The pushing activation led to the selective dissociation of the adduct by retro-DA with 58% conversion. In contrast, the pulling activation of the same adduct, with polymers attached to the oxanorbornene and maleimide sides, respectively, proceeds *via* 2 competing reaction pathways: the expected retro-DA (27%), regenerating the furan and maleimide rings, and the heterolytic scission of the C–N bond (20%), producing maleimide and trityl alcohol derivatives after reaction with water. CoGEF calculations revealed that during pushing activation, the maleimide is pushed away from the macrocycle, resulting in a twisting of the maleimide ring and the elongation of the C–C bond facing the macrocycle (a in Fig. 21). Pushing actuation does not induce any deformation in the upper part of the mechanophore, leaving the C–N bond (b in Fig. 21) unaffected. During pulling activation, tension is instead propagated along the vector connecting the two pulling points, leading to the activation of the most compliant bonds along the way. In this case, both bonds a and b are activated, with bond a elongating slightly faster than bond b, which explains the predicted retro-DA pathway and its preponderance during sonication. This work showed how the rotaxane actuator led to both a more efficient and selective activation than pulling the same mechanophore.

**Fig. 22:**
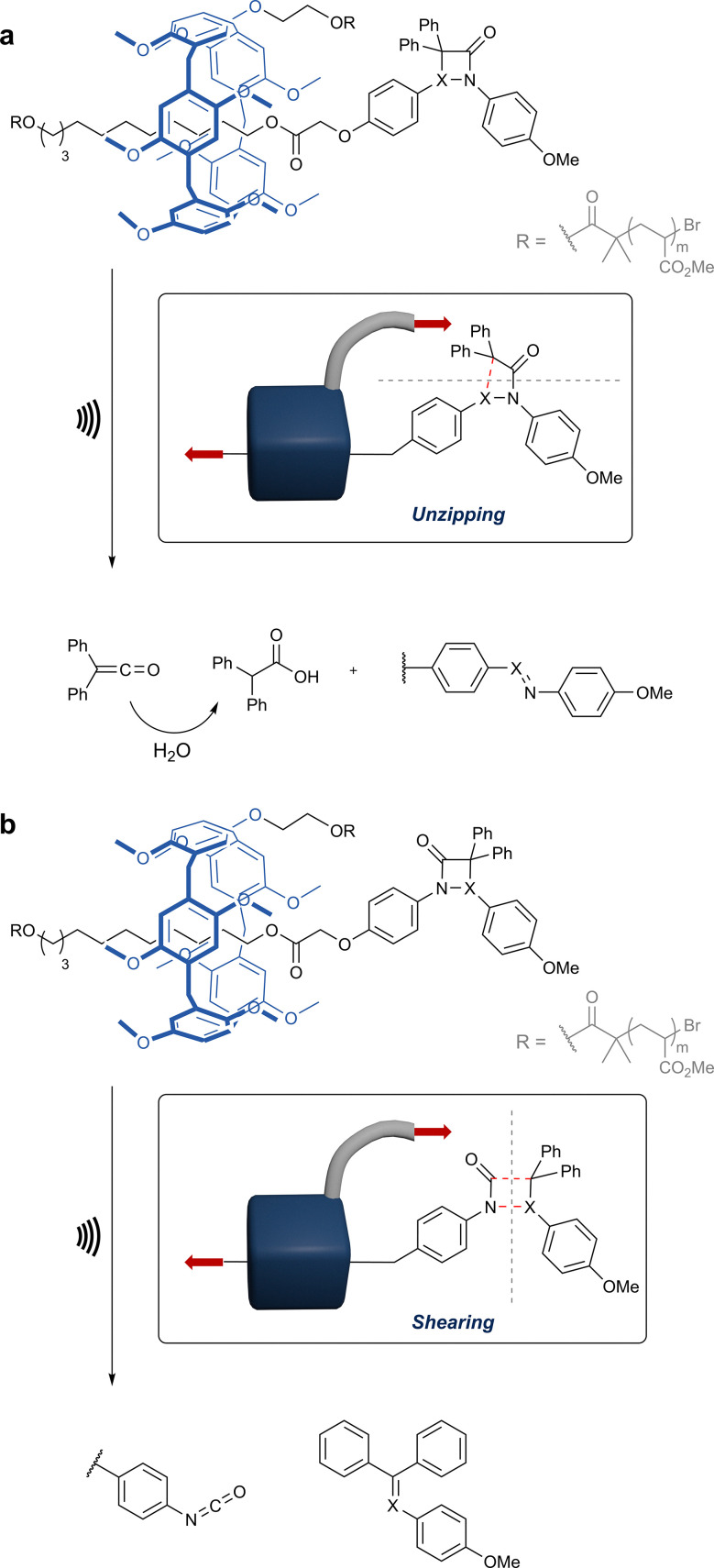
Orthogonal bond scission of a 4-membered ring upon pushing force of a rotaxane actuator. (a) Unzipping behaviour in the *cis*-isomer. (b) Shearing behaviour in the *trans*-isomer. Conditions: US (20 kHz, 13.0 W cm^−2^, 1 s ON/1 s OFF), THF/H_2_O: 9 : 1, 5–10 °C, 90 min. Red arrows indicate the direction of force, and scissile bonds are shown as red dashed lines. X = N or CH.

It was later shown that this pushing activation can be used to promote the scission of various bonds and release different species from the same mechanophore depending on its orientation within the rotaxane.^120^ A similar rotaxane to the one described above was stoppered by either diazetidinone^121^ or β-lactam^122^ mechanophores appended to a bulky gem-diphenyl (gDP) group. Depending on whether the gDP group was oriented towards (*cis*-) or away from (*trans*-) the macrocycle, the rotaxane actuator causes an unzipping or shearing dissociation pathway, respectively. In the *cis*-isomer, the macrocycle immediately pushes against the gDP group, resulting in the elongation the C–X bond and release of a diphenyl ketene (Fig. 22a). In the *trans*-isomer, the macrocycle is able to pass over the 4-membered ring of the mechanophore before reaching both the gDP and methoxyphenyl units concomitantly, resulting in the elongation of both bonds leading to the release of triphenylethylene or a triphenylmethanimine derivative (Fig. 22b). This work introduces the ability to generate a variety of functional molecules, here an acid, a dye, and a drug precursor, amongst others, from the same core structure without having to completely redesign the mechanophore.

**Fig. 23:**
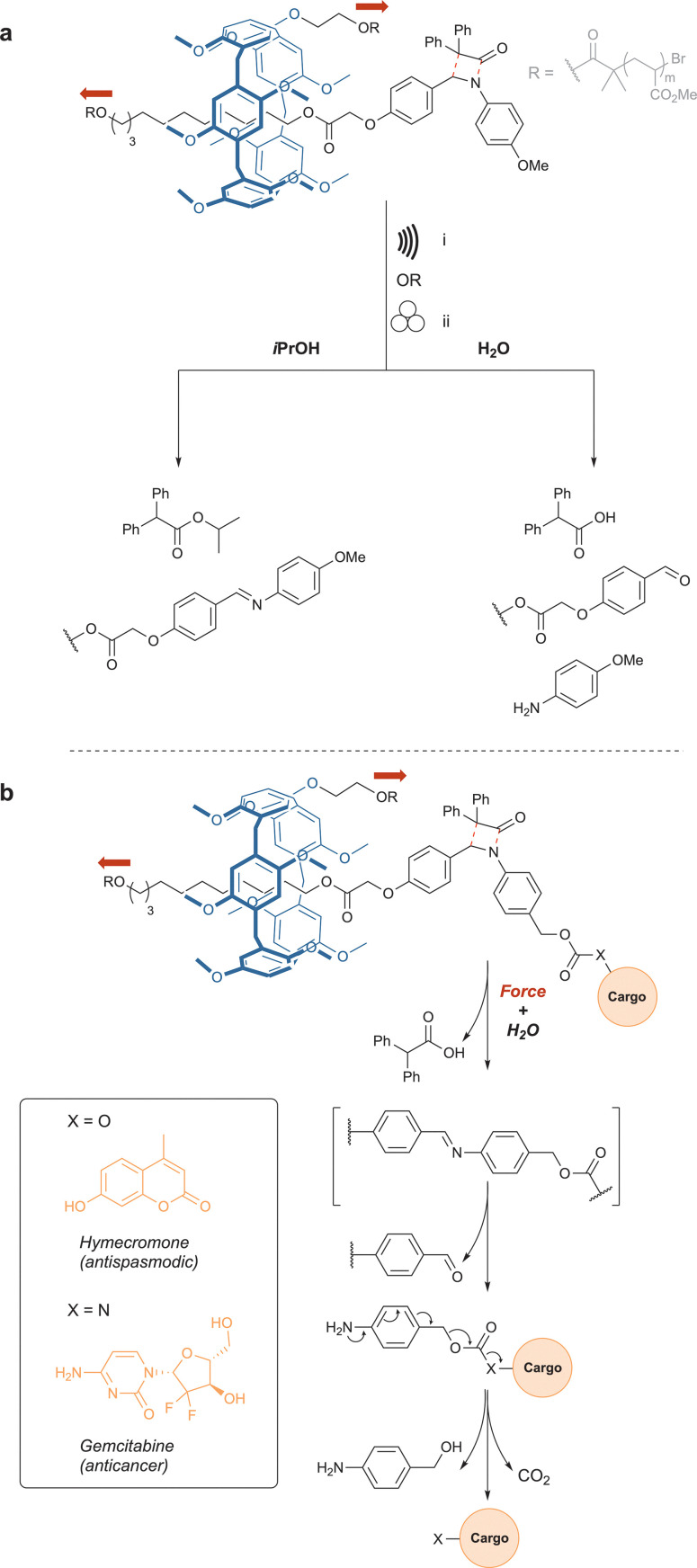
Logic gated release of a dual payload from a β-lactam mechanophore with a rotaxane actuator (a) and its use with a self-immolative linker to release various cargo molecules (b). Conditions (i) US (20 kHz, 13.0 W cm^−2^, 1 s ON/1 s OFF), CD_3_CN/iPrOH or CD_3_CN/H_2_O 9 : 1, 5–10 °C, 105 min. (ii) Cryomilling (30 kHz), 15 min. Red arrows indicate the direction of force, and scissile bonds are shown as red dashed lines.

The activation of the *cis*-isomer of the rotaxane bearing the β-lactam mechanophore revealed an imine functionality on the end of the axle. This device was later harnessed to allow for the release of a dual payload in response to two stimuli, a behaviour reminiscent of a logic AND gate (Fig. 23a).^123^ When the sonication was carried out in the presence of *i*PrOH, the imine moiety remains intact, while imine hydrolysis was observed in the presence of H_2_O. This release was achieved to a similar extent in both solution and bulk with similar efficiency up to 68%. By linking the β-lactam core to a cargo molecule through a self-immolative *para*-aminobenzyloxycarbonyl spacer (Fig. 23b), the release of various cargoes could be controlled by the necessity of two different stimuli, resulting in the release of two different drug molecules (hymecromone and gemcitabine). This dual-payload approach demonstrates the ability to precisely control the release of cargo molecules, with the requirement of two subsequent stimuli offering the possibility to control the timing and position of the released cargo.

**Fig. 24:**
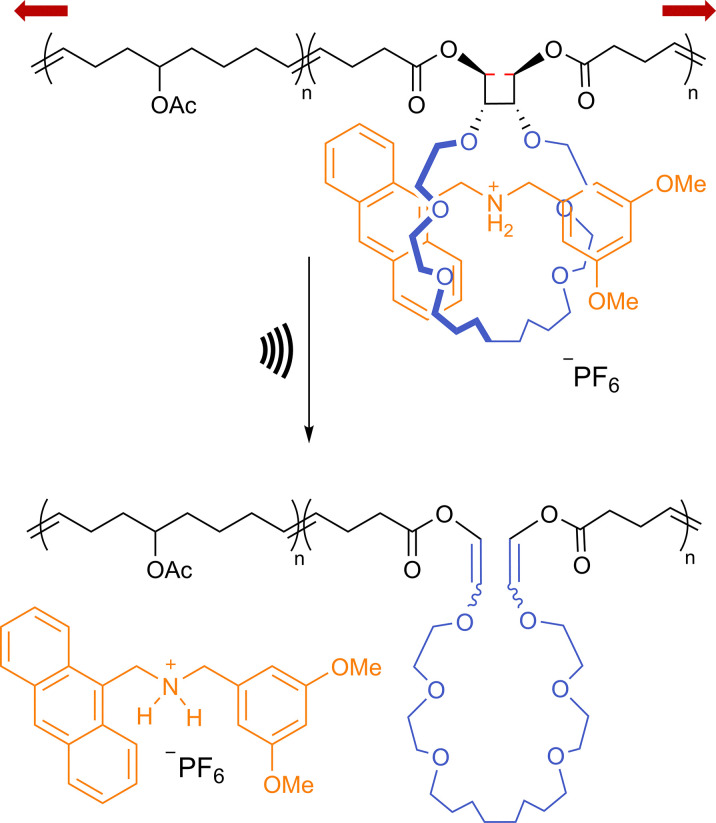
Force-promoted axle-release rotaxane containing a cyclobutane mechanophore in the macrocycle. (i) US (20 kHz, 0.92 W cm^−2^, 1 s ON/1 s OFF), THF, 6–9 °C, 120 min. Red arrows indicate the direction of force, and the first scissile bond is shown as red dashed line.

In addition to mechanical bond dissociation by pulling component parts against each other, fragments of the architecture can also be released by directly stretching a single structural element. Studies have shown how application of force across a short path in a macrocycle can result in covalent bond scission.^106,124^ This cleavage mechanism was later harnessed by Yang and Xia to release the axle component from a rotaxane (Fig. 24).^125^ A 24-membered crown ether macrocycle, containing a cyclobutane mechanophore, was cyclised around a dibenzylammonium axle. The axle contained an anthracene stopper which exhibits strong fluorescence, allowing the determination of the extent of axle release. Copolymers containing 4% and 20% of the rotaxane mechanophore were activated by ultrasonication, causing the cleavage of the cyclobutane unit in the macrocycle and the release of the axle. The decrease in the anthracene ^1^H NMR spectroscopy and UV-vis spectroscopy signals in the resulting polymer was used to determine the extent of axle release as being 39%. In the solid state, multiple compression cycles of a crosslinked polyurethane induced 1.8% of mechanophore activation. The above studies highlight different possible strategies for use of the rotaxane architecture for small molecule release, providing a platform for more advanced designs in the future.

#### 3.2.3 Remodelling mechanically interlocked molecules

**Fig. 25:**
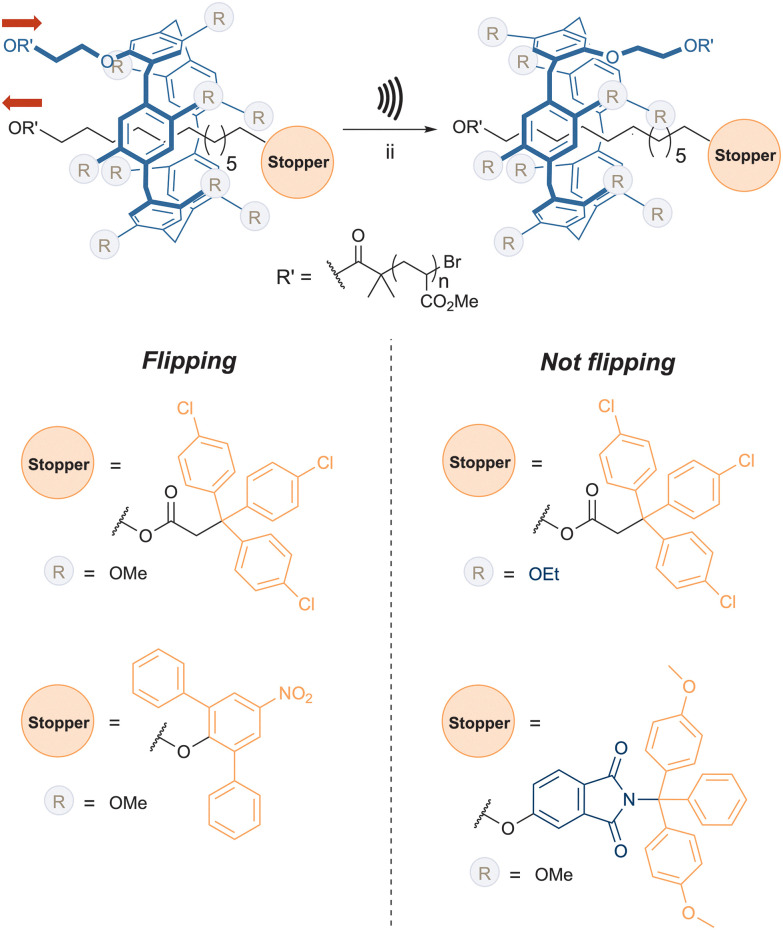
Force-promoted ring flipping in a threaded P5 macrocycle. Conditions (i) US (20 kHz, 13 W cm^−2^, 1 s ON/1 s OFF), MeCN/H_2_O: 50 : 1, 5–10 °C, 90 min. Red arrows indicate the direction of force.

In the previous examples, rotaxanes were used as force actuators to promote the activation of mechanophores attached to the axle. However, mechanical force can also be used to remodel the structure of the rotaxane itself. This concept was recently demonstrated by the De Bo group, with the mechanical flipping of one of the aromatic groups flanking a threaded pillar[5]arene to obtain an elusive conformation of this macrocycle where one hydroquinone ring is pointing in a different direction than the other 4.^98^ To achieve this, the *cis*-isomer of a P5-based rotaxane, in which the actuating polymers are found on the same side of the rotaxane structure, was appended with a bulky tris(4-chlorophenyl)methyl group (Fig. 25). On the application of mechanical force, the hydroquinone ring on which the polymer is attached is rotated around its internal axis, resulting in the P5 macrocycle with a single flipped ring. The ring inversion only occurs when the contact between the two subcomponents forces the alkoxy substituent inside of the macrocycle's cavity. This flipping behaviour was observed as the major pathway (42%) followed by unstoppering (38%). It was found that the flipping behaviour could be controlled by changing the nature of the stopper, the axle, or the macrocycle itself, as increasing the steric bulk of each component prevents the full rotation of the ring and can lessen or suppress the flipping. Interestingly, the rotaxanes that undergo flipping also result in greater unstoppering and are less susceptible to scission in the PMA arms. This work shows how careful design and control of mechanical bonds can introduce subtle ways to control the dissociation of rotaxanes and dissipate mechanical energy, leading to the development of more advanced mechanically interlocked materials. It also shows how mechanical force can be used to assemble intricate supramolecular architectures otherwise inaccessible.

**Fig. 26:**
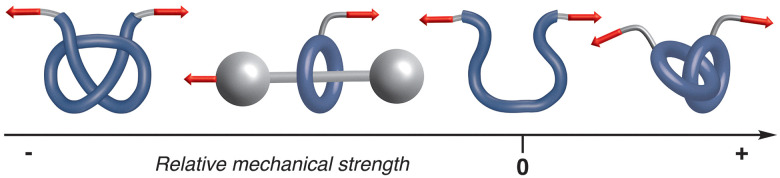
Relative mechanical strength of the three canonical interlocked structures: catenanes, rotaxanes, and knots.

### 3.3 Key insights and outlook from mechanochemistry

Interlocked molecules display contrasting behaviours under tension. At low force, elongation can induce a variety of geometry changes expected from their propensity for large amplitude movements: catenane is extended by ring rotation, the macrocycle of a rotaxane is shuttled from its station towards the end of the axle, and a knot is tightened. In effect, these structures are pulled away from their equilibrium (co-)conformation. This has proved useful for the assembly of rotaxane-based force sensors when the displacement of the macrocycle induces a change in photophysical properties. As mechanical force is inherently directional, it could be used to operate more advanced molecular machines.

The response of the three canonical mechanical bonds diverges at higher forces where covalent bond scission becomes possible (Fig. 26). A catenane acts as a force-protecting group due to the ability of macrocycles to spread tensile stress equally around their circumference, while leaving linear segments of the polymer to take much of the load. Consequently, catenanes are unlikely to break under tension. In contrast, rotaxanes and knots are more susceptible to breakage under tension than their non-interlocked counterparts. This comes from the fact that rings under tension, being either the macrocycle in a rotaxane or a loop of a knot, can induce a substantial amount of molecular deformation (*e.g.* elongation and torsion) to the molecular segments threaded inside their cavity. This phenomenon indicates that polymer materials containing such structures could fail prematurely at high load. This finding is particularly relevant in slide-ring networks (see Section 4) where mobile crosslinks enable the dissipation of mechanical energy at a low level of deformation, but these same crosslinks could accelerate the failure of the material at high levels of deformation.

Nevertheless, the ability of a macrocycle to induce the selective scission of a covalent bond, which is otherwise unreactive, in the axle of a rotaxane shows great potential for its use as a force actuator. Indeed, rotaxanes have been shown to be excellent actuators for the activation of mechanophores. Notably, in force-controlled release applications, the rotaxane architecture allows for the repetitive and sequential activation of mechanophores appended to the axle, as one of the actuating polymers is not covalently attached to the mechanophore. This strategy has enabled the release of a variety of functional molecules, sometimes following a reaction pathway accessible only through the rotaxane pushing activation mode. Moving forward, the use of mechanical force for the remodelling of interlocked molecules could provide new ways to assemble challenging supramolecular architectures and deliver new molecular mechanisms for the dissipation of mechanical energy.

## 4. Mechanically interlocked polymer networks

The architecture of a polymer material, such as linear, branched, or cyclic is an essential feature to control to determine its properties. In the last two decades or so, a new class of polymer architectures has been developed: mechanically interlocked polymers (MIPs), which are polymers containing mechanical bonds. The MIPs can be classified into four main categories: the polyrotaxanes,^126^ the polycatenanes,^127^ the daisy-chain polymers,^128^ and the slide-ring materials (SRMs).^129^ All these MIP types have been synthesized and studied at different degrees of depth, revealing the impact of the mechanical bonds on a large range of material properties in bulk or solution states such as crystallization, glass transition temperature (*T*_g_), solubility, bio-compatibility, electrical conduction, *etc.*^130^ In this section, we will specifically focus on how the mechanical link directly affects the mechanical and rheological properties of the materials, *i.e.*, beyond the indirect impacts associated with some of the above sited properties such as *T*_g_ or crystallization. In this context, the most studied MIP type is by far the slide-ring materials.^129^ In contrast to the systems discussed in the previous sections of this review, where a rather limited number of studies have been reported, many studies have been reported on slide-ring materials. This section will thus adopt a less detailed, more general, perspective for the discussion. Since it is beyond the scope of this review to give a comprehensive overview of the literature on SRMs, we will limit ourselves to selected key features, to give to the reader a general impression of the properties and potential of these interesting materials.

**Fig. 27:**
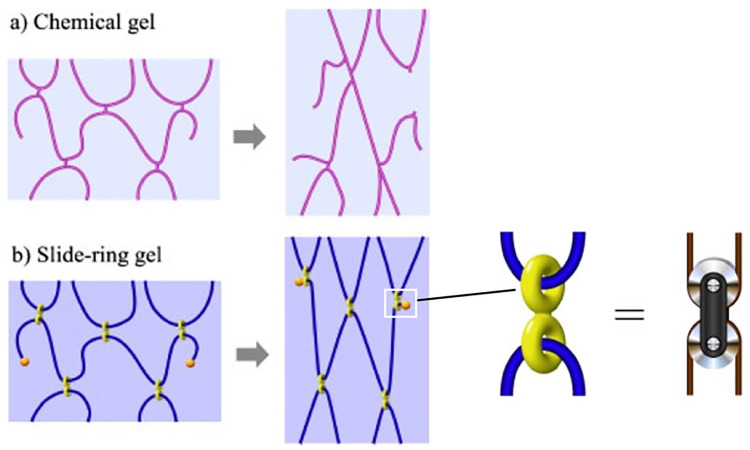
Schematic comparison between chemical (a) and slide-ring (b) gels with freely movable figure-of-eight crosslinks acting like pulleys. Adapted from ref. 133 with permission from the Society of Polymer Science, Japan.

Polymer networks are three-dimensionally crosslinked macromolecules and are called gels when the network is swollen by a liquid. The networks can be based on covalent or non-covalent crosslinks. These networks have very different properties and the advantages of one type are often the drawbacks of the other type. An alternative to these classical approaches is to use mechanical links as crosslinks, giving rise to the so-called slide-ring materials.^129^ The crosslinks in these materials are made of a rotaxane-like motif, and this results in crosslinks having the strength of covalent bonds (a covalent bond must be broken to break the network), while, at the same time, the rings can move (slide) along the polymer chains. These movable crosslinks can equalise the tension in the whole network when a force is applied, in a similar way as to how a pulley works, as opposed to chemical networks where the inhomogeneous crosslinking concentrates the stress on the short network strands, leading to early mechanical rupture (Fig. 27).^131,132^

This behaviour of SRMs is called the pulley effect and is the basis of all the interesting characteristics of these materials. For example, some SRMs may exhibit very large extensibility, up to 24 times their initial length without hysteresis, or can absorb solvents up to 24 000 times their initial dry mass.^133^ The SRMs have been mainly studied in the swollen (gel) state and are then called slide-ring gels (SRGs). SRMs of different architectures have been reported, but the most common system is by far the one based on polyrotaxanes made of (functionalised) cyclodextrins threaded on a poly(ethylene oxide) chain.^129^ The rings are then linked two-by-two to obtain figure-of-eight shaped crosslinks. Instead of directly linking the rings of the polyrotaxanes, the rings can be first functionalized by polymerizable groups or polymer chains, and the SRMs are then obtained by using these functionalised polyrotaxanes as a crosslinker for another polymer.^134–137^ Other architectures, not based on polyrotaxanes and cyclodextrins, have been reported,^138^ for example, using a crown-ether based rotaxane crosslinker bearing a vinyl group on the ring and at one end of the axle.^139^ However, an important point to keep in mind with these alternative strategies is the length available for the sliding of the rings, which is a crucial point as will be discussed later. Some of the reported architectures using rotaxane derived crosslinkers indeed exhibit only a small sliding distance and can thus barely be considered as SRMs. The above systems usually contain a rather limited amount of mechanical bonds, but other systems have been reported with a high density of mechanical bonds. In such materials, the dense mechanical bonds exhibit an integration and amplification mechanism, in contrast to SRMs that rely on the pulley effect. The mechanical bonds act as individual functional units that respond to applied forces, and their combined dynamics are integrated and amplified into the resultant macroscopic mechanical properties.^140^

As a result of the pulley effect, the mechanical behaviour of the SRGs is different compared to that of both physical gels and chemical gels. The stress–strain curve of the physical gels under large deformation usually shows a J-shape with a considerable amount of hysteresis. Due to the low structural memory, on deformation, the physical gels dissipate a part of the applied energy by breaking some of the weak bonds, which can then reform when the deformation is removed, resulting in hysteresis in the strain–stress curves. In contrast, the chemical gels exhibit a stress–strain curve with an S-shape and shows no hysteresis due to the stable covalent crosslinks. The slide-ring gels, on the other hand, display a J-shaped stress–strain curve similar to the physical gels, which indicates that slide-ring gels are soft with no elastic instabilities, but do not show any hysteresis, which means that the sliding motion is reversible and that these gels have a structural memory similar to the chemical gels.^133^

**Fig. 28:**
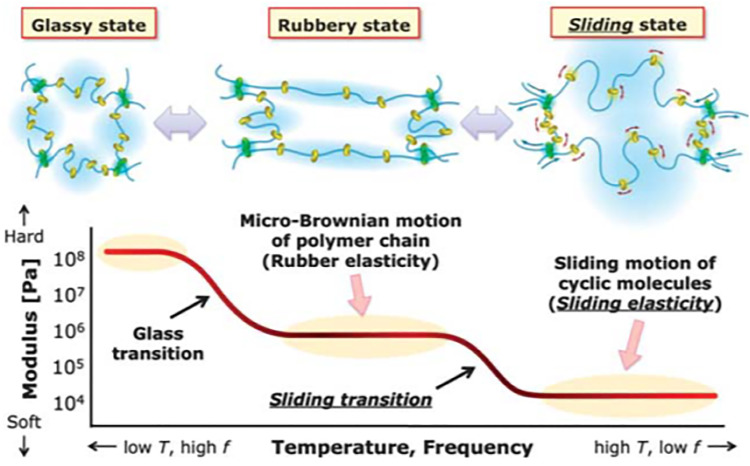
General rheological behaviour of slide-ring gels showing the different plateau moduli and the transitions between them. Adapted from ref. 132 with permission from the Society of Polymer Science, Japan.

Studies performed on SRGs have shown that the mobility of the crosslinks and the configurational entropy of the free (un-crosslinked) rings are two specific mechanisms that have to be taken into account to explain the properties of SRGs, in addition to the conformational entropy of the segments trapped between fixed crosslinks, which is present in classical polymer networks.^141–143^ A first impact of ring mobility is lower Young's modulus of SRGs compared to classical gels with fixed crosslinks. Models have shown that this behaviour is related to the available distance for ring sliding and to the ability of the system to remove the constraints and minimize its free energy.^142–144^ Ring mobility also impacts the viscoelastic behaviour of SRGs.^141,145^ The viscoelastic behaviour of chemical gels, and similarly of rubbers, is characterized by a finite equilibrium modulus which never relaxes. In contrast, like liquids, physical gels have no finite equilibrium modulus, and only a rubbery plateau is observed, which subsequently relaxes by the reptation mechanism and leads to completely disentangled chains. It is interesting to see that the slide-ring gels behave like an entangled polymer due to the presence of slidable crosslinks. However, the most significant difference is that the bulky stoppers at each chain-end of the polymer axle prevent the complete relaxation of the network, which is a situation similar to chemical gels. The combination of these features yields two distinct plateaus in the storage modulus after the glass transition: one at high frequency and the other at low frequency (Fig. 28).^132,141,145^ The origin of the high frequency plateau (rubbery plateau) is explained, as for classical gels, by the entropic elasticity of the polymer strands between two crosslinks where the polymer chains do not slide at all. The second, low frequency, plateau, called the “sliding state”, is linked to the sliding motion of the polymer chains through the cyclic crosslinks, but its exact origin and the underlying mechanisms are still unclear. The effective range over which the ring crosslinks can slide also strongly influences the SRG properties.^133,141–143,146^ This range can be modified by changing the molar mass of the polymeric axle, which improves the fracture energy, but other factors such as the crosslinking density and the aggregation of the rings, which depends on the solvent used, have to be considered since they limit ring mobility. More detailed discussions can be found in a recent review on the dynamics of ring-containing polymers.^147^

**Fig. 29:**
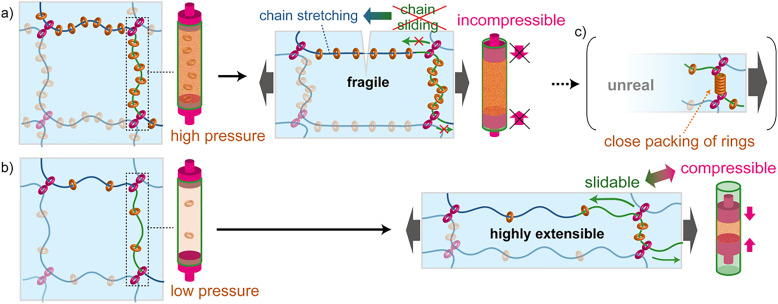
Schematic illustrations of the network structure of polyrotaxane gels with different ring densities: (a) large coverage and (b) low coverage. (c) The unreal close packing state under the maximum strain. Adapted from ref. 149 with permission from the Royal Society of Chemistry.

To fully explain the behaviours of SRGs, another parameter should be taken into account: the presence of free (uncrosslinked) rings along the polymeric axle.^148–151^ These free rings generate a translational entropy which counteracts the sliding of the crosslinks. An analogy could be made where the free rings between crosslinks are considered as gas molecules in a cylinder closed by movable pistons (Fig. 29).^149^ On deformation, the sliding of the crosslinks is hampered by the counter-pressure created by the free rings being “compressed”. This effect, called sliding elasticity, is especially important in gels with high ring coverage (defined as the percentage of the polymeric axle covered by the rings) as compared to the low ring coverage gels. As a result, the crosslink sliding becomes inactive even at low strain in high ring coverage gels, while remaining active in low ring coverage gels which display very high extensibility. A model combining the crosslink sliding and the sliding elasticity from the free rings has been proposed which can reproduce the observed behaviours of some SRGs such as the non-linear dependence of Young's modulus on crosslinking density.^133,143,144^ The modulus indeed increases with crosslinking density for small amount crosslinks, while it decreases for larger amounts. However, this evolution of the modulus is not always observed and depends on the characteristic of the SRGs such as ring coverage. Despite all these advances, the exact influence of ring entropy on the mechanical properties of SRGs is still unclear, and some discrepancies between theory and experimental results persist. Further studies and modelling are thus needed.

The mobility of the ring crosslinked is thus the key parameter influencing the SRG properties. This mobility depends, in turn, on several factors such as crosslink density, ring–axle interactions, and the distance available for ring sliding. The chemical composition and structure of the building blocks used to prepare the SRGs thus have a huge impact on the SRG properties. For example, as already mentioned above, SRGs prepared from low ring coverage polyrotaxanes generally exhibit better mechanical properties (higher extensibility and stress at break, better fatigue resistance, small residual strain, and increased fracture toughness) than at high coverage.^148–151^ The ring–axle interactions can be controlled either by changing the size of the rings^152,153^ or the “thickness” of the polymeric axle,^154^ or by using specific chemistry such as metal–ligand complexes holding initially the rings on a binding station and then removing the metal ions to free the rings,^155,156^ or light responsive azobenzenes to tune the interactions with the cyclodextrins.^157^ An increase in ring–axle interactions increases the force required to pull the rings along the axle or, in other words, increases the friction associated with ring sliding and leads to a larger fracture stress while not influencing the strain at break.

Understanding the structure–property relationships is thus essential to further develop and exploit these unique materials. Fully understanding the elasticity of classical networks is already challenging because of the presence of defects such as dangling ends or loops, but it is even more difficult in SRGs because of the additional elasticity mechanisms discussed above as well as the influence of some parameters such as interactions between the rings and/or their aggregation that can be difficult to control.^158,159^

**Fig. 30:**
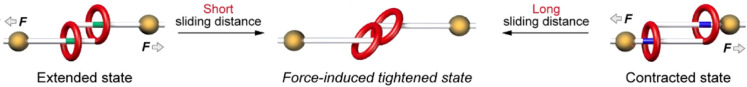
Schematic drawing of two bistable [c2]daisy chains each in a defined state (extended or contracted) used to prepare polymer networks. Adapted from ref. 162 with permission from the American Chemical Society, copyright 2023.

As demonstrated above, SRMs are clearly the most studied MIPs, but other systems also show promising properties. For example, dimeric cyclic daisy chains, *i.e.*, [c2]daisy chains, are especially interesting because they allow the components to slide past each other, contracting or expanding the interlocked motif, in a motion reminiscent of the one occurring in sarcomere muscles.^128,160,161^ However, as for the SRMs, the elucidation of the relationships between molecular motions and macroscopic (mechanical) properties is challenging. To progress in this direction, a recent study reported on two networks each made from bistable [c2]daisy chains in a defined state: one already in the extended state and the other in the contracted state (Fig. 30).^162^ The authors showed that the network based on extended [c2]daisy chains exhibits higher strength and good creep resistance, while the network based on the contracted form exhibits enhanced ductility and energy dissipation, thanks to the long-range sliding motion.

Slide-ring materials thus represent a specific class of polymeric systems in which topological design, rather than conventional covalent architecture, governs macroscopic properties. The pulley effect, arising from mobile cross-linking points in polyrotaxanes, is the underlying key concept, enabling the unusual decoupling of stress and deformation and conferring toughness, extensibility, and recovery to the material. Looking forward, a central challenge, but also an opportunity, lies in establishing a predictive framework that connects nanoscale molecular mobility to bulk mechanical properties. While the dual entropy contributions of polymer chains and cyclic components offer an interesting design space, cross-linking still imposes constraints that limit the full expression of ring dynamics. Future efforts should therefore focus on molecular architectures that preserve or actively regulate this mobility, potentially leveraging stimuli-responsive polyrotaxanes to create adaptive or programmable materials.

Moreover, the prevalent polyethylene glycol/α-cyclodextrin platform suffers from limited thermal stability and non-negligible energy barriers to ring sliding. The development of alternative axle and macrocycle chemistries with enhanced thermal robustness and smoother translational motion will be crucial for extending SRM applications into more demanding environments. Overall, the future of SRMs will thus depend on advances in molecular design, mechanistic understanding, and multiscale integration.

## 5. General discussion

MIMs and polymers, encompassing architectures such as rotaxanes, catenanes, knots and slide-ring systems, have emerged as a powerful platform for interrogating motion, force transduction, and adaptive behaviour at the molecular scale. Notwithstanding remarkable conceptual and synthetic progress, the field continues to grapple with a set of interrelated limitations that constrain both mechanistic interpretation and the translation of molecular design into functional materials.

A central experimental challenge lies in the intrinsic signal-to-noise limitations of single-molecule force spectroscopy when probing sub-nanometre displacements. Many of the most chemically and mechanically informative processes in interlocked systems—such as ring shuttling across small barriers or local conformational rearrangements—occur over length scales that approach the thermal noise floor. Under such conditions, thermal fluctuations and instrumental drift collectively obscure the signatures of discrete molecular events. Consequently, the assignment of force–extension features to specific structural transitions is sometimes ambiguous, and the extraction of quantitative parameters such as barrier heights or transition distances becomes highly model-dependent. Although advances in instrumental stability and data analysis have improved precision, the fundamental kT-scale fluctuations impose a limit that cannot be entirely eliminated, rendering the interpretation of sub-nanometre events inherently probabilistic.

A further conceptual and practical difficulty arises in the quantification of force-dependent free-energy landscapes. SMFS experiments typically probe systems under non-equilibrium conditions, complicating the direct application of equilibrium statistical mechanics. Analytical frameworks such as the Bell–Evans or Dudko–Hummer–Szabo models provide useful approximations, yet they rely on assumptions regarding the barrier shape and reaction coordinates that are rarely validated independently. Consequently, extracted parameters can vary significantly depending on the chosen model. The situation is exacerbated by limited sampling, as experimental datasets often comprise relatively small numbers of rupture or transition events, and by uncertainties in force calibration, which propagate nonlinearly into derived energetic quantities. Non-equilibrium work relations, including Jarzynski's equality and Crooks’ fluctuation theorem, offer, in principle, a route to reconstruct free-energy profiles; however, their practical application demands pulling-relaxing experiments that are challenging to perform in many experimental settings.

Other limitations are persistent discrepancies between simulations and measurements. Molecular dynamics and coarse-grained approaches have become indispensable for rationalizing the behaviour of interlocked systems under force; however, their predictive power remains uneven. Standard force fields are seldom parameterized for the unusual topologies and highly strained geometries characteristic of MIMs, particularly under large external forces. Moreover, the accessible timescales in simulations—typically spanning nanoseconds to microseconds—remain several orders of magnitude shorter than those probed experimentally, leading to an incomplete sampling of rare events and slow barrier crossings. Differences in pulling protocols further complicate comparisons, as constant-velocity or constant-force conditions *in silico* do not map straightforwardly onto experimental implementations. In addition, simplified treatments of the environment, including implicit solvent models, often neglect hydrodynamic effects that can significantly influence molecular mobility. As a result, simulations frequently capture qualitative trends but fail to reproduce quantitative observables such as transition rates or free-energy barriers, raising important questions regarding their use in predictive design.

From a synthetic perspective, the preparation of interlocked molecules remains a persistent bottleneck that limits the diversity and scalability of mechanically interlocked architectures. This limitation is further complicated by the additional structural complexity required to interface a mechanical bond with an external force (*i.e.* connect it to a polymer) and to apply this force in a controlled fashion. Some of these challenges can be alleviated using small, symmetrical macrocycles (*e.g.* crown ethers, pillararenes, and cyclodextrins), which remove the need for bespoke building blocks (such as stoppers and macrocycles) that otherwise significantly lengthen the synthesis of interlocked molecules. At the same time, new strategies continue to emerge which enable the efficient synthesis of interlocked molecules from a broader range of building blocks.

In polymeric systems incorporating mechanical bonds, such as slide-ring networks, additional layers of complexity emerge from structural and mechanical heterogeneity. Variations in threading number, ring distribution along polymer backbones, and network topology give rise to broad distributions of mechanical responses. This heterogeneity complicates the establishment of clear structure–property relationships and hinders the extrapolation of single-molecule insights to macroscopic behaviour. Moreover, subtle differences in molecular conformation, attachment geometry, or local environment can lead to significant variability even among nominally identical interlocked species, further blurring the connection between the designed structure and the observed function.

Control over the directionality and efficiency of motion within MIMs remains another unresolved challenge. Although numerous systems exhibit stimulus-responsive shuttling or rotation, these processes are often dominated by Brownian motion, with energy inputs leading to stochastic rather than deterministic behaviour. Achieving sustained, directional motion that can perform useful work with high fidelity requires precise coupling between energy input and mechanical output, a goal that remains only partially realized. This limitation is particularly significant in the context of developing molecular machines capable of operating under load or within complex environments.

The long-term mechanical stability of interlocked systems under repeated cycling also warrants careful consideration. For example, the mechanical susceptibility of rotaxanes could be detrimental to their use as dynamic crosslinkers in slide-ring materials. Additionally, the covalent components that define the architecture—such as axles, stoppers, or polymer backbones—may undergo fatigue, chemical degradation, or irreversible conformational changes over time. In polymeric materials, repeated deformation can lead to network rearrangement, loss of threading, or other forms of structural deterioration, raising questions about durability in practical applications.

Finally, the integration of mechanically interlocked motifs into functional materials and devices presents substantial challenges in terms of scalability, organization, and force transmission across length scales. Aligning and orienting interlocked units within bulk materials, ensuring efficient coupling between molecular motion and macroscopic properties, and maintaining compatibility with conventional processing techniques all remain nontrivial tasks. Bridging the gap between well-defined molecular behaviour and emergent material function is therefore an ongoing area of active investigation.

Taken together, these limitations underscore the intrinsically multidisciplinary nature of the field. Progress will depend on the continued co-development of high-resolution experimental techniques, more accurate and transferable simulation methodologies, and scalable synthetic strategies. Equally important will be the refinement of theoretical frameworks capable of rigorously describing non-equilibrium processes under force. Addressing these challenges is essential for advancing mechanically interlocked molecules and polymers from sophisticated model systems toward reliable, engineerable components in next-generation functional materials.

## 6. Conclusions

Mechanical forces are fundamental to almost every key biological process. The widespread use of molecular machines in nature has inspired scientists who have tried to design synthetic molecular systems with architectures where triggered positional changes of sub-molecular components occur. Sixty years after chemists carried out the first directed synthesis of a catenane, numerous strategies now exist for the synthesis of MIMs. The appeal of these molecules lies in the possibility of controlled motions of relatively large amplitude in which one component moves with respect to another, potentially generating net directional forces. Artificial molecular machines that can effectively generate mechanical work from a directional force to perform net tasks and polymer materials with unprecedented mechanochemical properties are emerging, and the quick pace of progress in the field should give rise to more advanced systems.

In the past decade, the tremendous experimental challenges to effectively interface small synthetic molecules with single-molecule force spectroscopy techniques have been mastered and a number of MIMs could be successfully investigated, one at a time. The difficulty stems from the development of proper tools and preparation of appropriate molecules because the amplitude of the motions involved is extremely small compared with those observed in larger biological systems or polymeric structures, particularly when monitoring minute structural changes under Brownian fluctuations. Successful attempts have enabled the direct measurement of forces involved in and/or work performed by catenanes and rotaxane-based structures, as well as knotted molecules. They have also allowed us to show that the performances of these artificial structures often surpass those of their biological counterparts. Additionally, single-molecule force spectroscopy experiments have provided unprecedented insights into their operation and dynamics.

Polymer mechanochemistry using mechanical bonds has shown how different interlocked structures have varying impacts on the stability of their constituent covalent bonds, with rotaxanes and knots accelerating bond scission, while catenanes can behave as a mechanochemical protecting group. Now the unique mechanochemical reactivity of interlocked molecules is exploited to create force-responsive molecular devices and materials. The shuttling properties of the rotaxane have been harnessed to assemble mechanochromic devices used to detect different levels of deformation or damage in a material. Rotaxanes are also excellent force actuators, and they have been used to create molecular machines able to release various cargo molecules or elicit new mechanochemical reactions in response to external force. As the complexity of mechanical bonds continues to increase, new designs will allow unique force-promoted reactivity, leading to further advances in the design of future responsive devices and materials.

Finally, mechanically interlocked polymer networks, in which mechanical bonds act as individual functional units that respond to applied forces and their combined dynamics are integrated and amplified into the resultant macroscopic mechanical properties, are clearly exciting and promising systems both on a fundamental and application point view. However, the development of the field is still limited by difficulties of chemical synthesis, especially the synthesis of macrocycles which is very often low-yielding. Moreover, many of the reported systems are complexified by uncontrolled parameters such as, *e.g.*, network defects, ring interaction or aggregation, or inhomogeneous composition, which can hamper the quantitative understanding of the relationships between the molecular scale motions and the macroscopic properties. The development of more controlled model systems, together with molecular simulations, would certainly help the field progress.

## Conflicts of interest

There are no conflicts to declare.

## Data Availability

No primary research results, software or code have been included and no new data were generated or analysed as part of this review.
